# AI-based analysis of key predictors of CO_2_ emissions across income groups

**DOI:** 10.1016/j.isci.2026.116976

**Published:** 2026-07-30

**Authors:** Rihab Fannouch, Saïd Tounsi

**Affiliations:** 1 Laboratory of Applied Economics, Mohammed V University, Rabat, Morocco

**Keywords:** CO2 emissions, deep learning, macroeconomic drivers, income groups, feature importance, climate change mitigation

## Abstract

This study examines how macroeconomic, energy, demographic, and trade factors are associated with CO_2_ emissions across country income groups using World Bank World development indicators (WDI) data (1990–2023). A set of deep learning models (multilayer perceptron (MLP), recurrent neural network (RNN), long short-term memory (LSTM)) is evaluated to capture non-linear patterns, and the MLP yields the best predictive accuracy. To interpret the main predictors underlying model performance, random forest (RF) feature-importance and correlation analyses are conducted, revealing systematic heterogeneity across development stages: In lower-income countries, emission patterns are more strongly linked to the energy mix and natural-resource endowments, whereas in middle- and high-income economies they are increasingly associated with trade exposure, fuel dependence, industrial activity, and demographic pressures. These findings should be interpreted as predictive associations rather than causal effects and support the design of differentiated mitigation policies aligned with countries’ structural characteristics, rather than uniform global targets, to improve both effectiveness and equity in emission reduction strategies.

## Highlights

• AI framework links macroeconomic, energy and trade factors to CO_2_ across incomes
• Deep learning (MLP) best predicts CO_2_; Random Forest ranks key predictors
• Low-income CO_2_ tied to energy mix; richer economies to trade and fuel dependence
• Income-specific predictors support differentiated mitigation policies

## Graphical abstract

**Figure:**
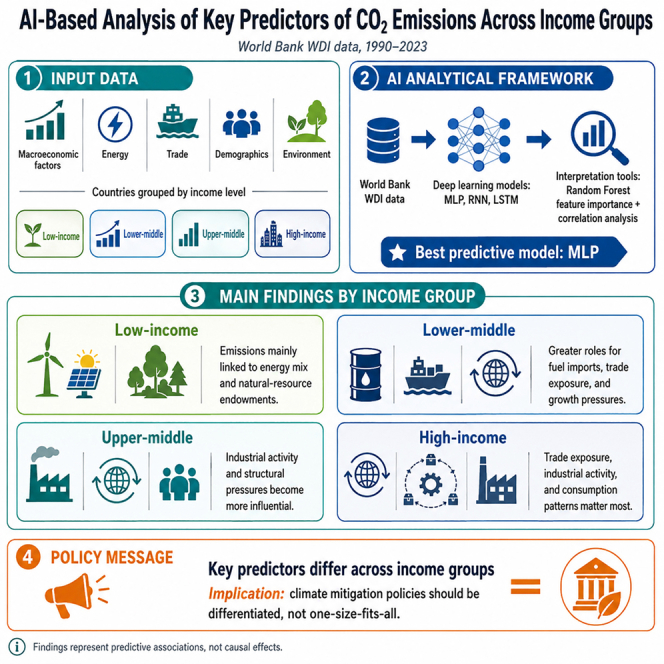

## Teaser

Pollution; Artificial intelligence; Economics

## Introduction

Climate change remains one of the most pressing and complex challenges of the twenty-first century, with carbon dioxide (CO_2_) emissions at the core of global warming and long-term ecological disruption. CO_2_ emissions are tightly linked to industrial activity, energy use, urbanization, and trade, making them a key indicator for assessing the sustainability of development pathways and the effectiveness of climate policies. As economies expand, particularly in emerging and developing countries, the tension between growth objectives and environmental constraints raises critical questions about how macroeconomic structures, energy systems, demographic dynamics, and trade patterns are associated with CO_2_ emissions trajectories.^1^

A vast literature has examined the relationship between economic development and environmental degradation, often through the lenses of the environmental Kuznets curve (EKC), the pollution haven and pollution halo hypotheses, and related frameworks.^2^ Early contributions documented a positive association between gross domestic product (GDP) growth and emissions in both advanced and developing economies, highlighting the environmental cost of industrialization and urban expansion.^3^ More recent work has broadened the analysis to include energy mix, trade openness, foreign direct investment, financial development, and institutional quality, showing that the growth-emissions nexus is mediated by the composition of output, the sources of energy, and the degree of integration into global markets.^4^ These findings have fueled ongoing debates about the conditions under which economic growth can be decoupled from carbon emissions and about the design of effective, context-specific mitigation strategies.

The challenge of balancing development and environmental protection is particularly acute for low- and middle-income countries, many of which are undergoing rapid structural transformation. In these contexts, growth is driven by a combination of capital accumulation, industrialization, demographic expansion, and trade integration, often under constraints related to technology, finance, and governance.^5^ At the same time, high-income economies face a different set of issues: Although many have begun to decouple economic activity from domestic emissions through energy efficiency improvements and environmental regulation, they remain central players in carbon-intensive global value chains and major consumers of goods with significant embodied emissions.^6^ These structural asymmetries across income groups underscore the importance of a comparative perspective on the predictors of CO_2_ emissions.

Recent evidence further highlights the unequal distribution of emissions relative to population. Low-income countries account for a small share of global CO_2_ emissions despite hosting a substantial proportion of the world’s population, whereas upper-middle- and high-income countries contribute a disproportionately large share of emissions relative to their demographic weight (World Bank, World development indicator (WDI)). Such imbalances raise concerns about equity, historical responsibility, and burden-sharing in international climate negotiations, and point to the need for mitigation strategies that are differentiated by level of development rather than uniform across countries.

Traditional econometric approaches have provided valuable insights into the emissions-growth nexus and related hypotheses, but they often rely on linear or pre-specified functional forms and may struggle to capture the non-linearities, interaction effects, and high-dimensional dependencies that characterize the relationship between CO_2_ emissions and their macroeconomic, energy, demographic, and trade determinants.^7^ In response to these limitations, a growing body of research has started to employ machine learning (ML) and deep learning (DL) techniques to forecast emissions and model complex environmental-economic relationships.^8^ These methods can flexibly approximate non-linear functions, uncover latent patterns, and rank the relative importance of predictors, thereby complementing traditional econometric tools. In this study, variable importance and correlation results are therefore interpreted as predictive associations rather than causal effects. However, most existing ML-based studies focus on single countries or specific regions, prioritize predictive accuracy over interpretability, or do not systematically compare key emission predictors across income groups.

This study addresses these gaps by adopting an AI-based framework to investigate the determinants of CO_2_ emissions across four World Bank income categories: low-, lower-middle-, upper-middle-, and high-income countries. Using recent data from the WDIs, the analysis considers a multidimensional set of explanatory variables that includes GDP per capita growth, foreign direct investment inflows, sectoral value added in agriculture, industry, and services, trade openness (exports and imports of goods and services), fuel imports, renewable energy consumption (REC), forest area, and population growth. This design allows for a joint assessment of macroeconomic performance, structural composition, energy dependency, demographic pressures, and natural capital in shaping emission trajectories across the development spectrum. Descriptive statistics reveal marked asymmetries between population shares and emission contributions for the four income groups, underscoring the disproportionate environmental responsibility of wealthier economies and the importance of equity-oriented, differentiated climate policies.

Against this background, the paper addresses three main research questions.

(i) To what extent can AI-based models identify the most influential macroeconomic, energy, demographic, and trade-related predictors of CO_2_ emissions within each income group?
(ii) How do these predictors differ across income categories, and what does this heterogeneity reveal about the structural underpinnings of emissions at different stages of development?
(iii) How can the resulting evidence inform differentiated climate mitigation policies that reconcile emission reduction goals with growth and development priorities?

This study makes three main contributions to the literature. First, it introduces a comparative AI-based analysis of key predictors of CO_2_ emissions across four income groups, combining deep learning models with random forest (RF) feature-importance and correlation analyses. This integrated approach exploits the predictive strengths of deep learning while retaining interpretability through ensemble-based variable importance measures. Second, by jointly considering macroeconomic, energy, demographic, and trade variables within a unified framework, the study provides a multidimensional characterization of emission predictors that goes beyond binary growth-emissions assessments and documents how the relative importance of each factor varies along the income distribution. Third, the findings are explicitly linked to climate policy design: The analysis highlights that emission predictors are heterogeneous across income groups and argues that effective and equitable mitigation strategies must be tailored to countries’ income levels, structural characteristics, and degrees of integration into the global economy, rather than relying on uniform global targets.

### Theoretical background

This study examines the determinants of CO_2_ emissions through a broad set of macroeconomic, demographic, energy, and environmental indicators, with an explicit focus on heterogeneity across income groups.

To frame the empirical analysis, this section outlines the key theoretical channels through which economic activity, structural change, globalization, and energy systems shape CO_2_ emissions, and highlights why these mechanisms may differ across income groups.

#### Scale, structure, and technology channels (macro-environment nexus)

At a fundamental level, emissions are shaped by three interacting forces: the scale of economic activity (how much output is produced), the structure of production (which sectors dominate), and the technology/energy mix used to generate output.^9^ In the scale channel, rising output tends to increase energy demand and industrial processes, which mechanically elevates emissions when the energy system remains carbon-intensive. In the structure channel, economies with a larger industrial value added share typically exhibit higher emissions intensity than service-oriented economies, while agriculture can have mixed effects depending on energy use, mechanization, and land-use pressures. In the technology channel, cleaner production methods, higher energy efficiency, and substitution from fossil fuels to renewable energy reduce the emissions content of each unit of output. This tripartite logic is consistent with the motivation of moving beyond GDP-only explanations by incorporating sectoral, energy, and environmental variables in the empirical framework.^2^

#### Energy-growth nexus and the role of renewables

Energy consumption is widely recognized as a central driver of emissions because fossil-fuel-based generation and use release CO_2_ directly. This mechanism underscores that energy is indispensable for economic activity while remaining environmentally costly.^10^

In contrast, REC is theoretically expected to reduce emissions by lowering the carbon intensity of energy supply. The strength of this mitigation effect depends on (a) the baseline fossil dependence, (b) the pace of renewable deployment relative to demand growth, and (c) institutional capacity (regulation, grid integration, enforcement).^11^ However, the energy-growth link is not automatic: inefficiencies, misallocation, and weak regulatory oversight can dampen the growth returns to energy use, thereby underscoring that governance and system quality condition both economic and emissions outcomes.

#### Income dynamics and the environmental Kuznets curve (EKC)

A key theoretical reference for income-environment interactions is the EKC hypothesis, which posits that environmental degradation may first rise with income (industrialization/scale effects) and later decline after an income threshold (technology upgrading, regulation, and preference shifts toward environmental quality).^12^ This study also considers common EKC extensions (including N-shaped trajectories) to reflect potentially non-linear development paths. In a four-income-group design, the EKC logic provides a direct theoretical basis for expecting different dominant key predictors across development stages (e.g., industrial expansion in middle-income groups versus consumption-based or outsourced emissions in high-income groups).

#### Globalization, trade, and “pollution shifting” mechanisms

International trade can affect emissions through multiple channels. Openness may increase emissions via scale effects (higher production for export), or reduce domestic emissions via composition and technology diffusion effects if trade enables cleaner technologies and shifts activity toward less carbon-intensive sectors. A complementary framework is the Pollution Haven Hypothesis (PHH), which suggests that pollution-intensive activities relocate toward countries with weaker regulation, potentially raising emissions in host economies. These mechanisms imply that the trade-emission relationship can vary systematically across development stages and regulatory capacities.

Importantly, in high-income economies, trade—especially imports—can be associated with a “consumption-based” footprint where part of the carbon burden is embodied abroad, while domestic emissions may decouple partially from consumption.^13^ This theoretical point motivates including both exports and imports as distinct predictors (rather than a single openness index).

#### Capital flows, industrial upgrading, and FDI

Foreign direct investment (FDI) can influence emissions ambiguously. On one hand, it may raise emissions if it expands carbon-intensive production capacity (“scale/dirty investment” channel). On the other, it can reduce emissions if it transfers cleaner technologies, managerial practices, and energy-efficient capital (“technology diffusion” channel).^14^ The net effect is therefore expected to vary with income level, regulatory strength, and the sectoral allocation of investment, which aligns with an income-group comparative strategy.^15^

#### Demographics, natural capital, and carbon sinks

Population growth affects emissions through demand expansion (housing, transport, electricity) and land-use pressures.^16^ At the same time, natural capital—captured by the forest area indicator—acts as a carbon sink and can mitigate net emissions when preserved and expanded.^17^ Conversely, deforestation can raise emissions both by releasing stored carbon and by reducing sequestration capacity.^18^ Including population growth and forest area therefore connects the analysis to basic demographic and ecological mechanisms.

#### Implications for an ML-based determinant analysis

The theoretical frameworks above motivate two methodological choices in this study: (1) using a multidimensional set of predictors (energy, trade, sectoral structure, demography, environment), and (2) allowing for non-linear and interaction effects that vary across income groups—patterns that traditional linear specifications may miss.^19^^,^^20^^,^^21^

Accordingly, the empirical results are interpreted as identifying predictive associations consistent with these mechanisms, rather than establishing causal effects.

### Empirical literature review

Environmental degradation continues to pose a critical obstacle to sustainable development, with carbon dioxide (CO_2_) emissions frequently used as a benchmark for gauging ecological harm.^22^ The main contributors to economic growth, population increase, technological innovation, and macroeconomic consolidation often intensify energy consumption and industrial processes, thereby accelerating resource depletion and contributing to climate disruption and biodiversity decline.^23^ These overlapping dynamics have prompted a growing body of research seeking to understand how development goals can be aligned with environmental preservation.

#### Growth-emissions nexus and GDP-Centered studies

In many cases, when GDP rises, CO_2_ emissions tend to increase as well, a trend that researchers have documented repeatedly over time. This link has been observed time and again. For instance, after studying countries such as China, Japan, and the United States, it was pointed out that emissions tend to increase as economies expand, and in some situations, these emissions might even help sustain that momentum.^24^ Similar outcomes were reported in Iran.^25^ Comparable evidence was found in Algeria,^26^ and the same pattern was noted across several African countries: Industrial growth seems to go hand in hand with environmental stress.^27^ These scattered findings echo a broader idea put forward by the Simon-Steinmann model, which views the link between economic progress and ecological impact as something deeply embedded in the way societies develop over time.

Panel data analyses employing cointegration techniques have further reinforced the centrality of GDP as a driver of emissions.^28^^,^^29^^,^^30^ However, the predominance of GDP and CO_2_ as the sole analytical variables has been increasingly criticized for oversimplifying complex environmental dynamics. Scholars now call for the integration of broader economic, sectoral, and institutional factors to enhance explanatory power and policy relevance.

#### Energy consumption and the role of renewable energy

Among the most influential determinants of CO_2_ emissions is energy consumption. Energy is indispensable for growth and social welfare, yet its production and use, especially from fossil fuels, remain major contributors to environmental degradation.^31^ Empirical studies confirm that higher energy consumption tends to elevate emissions, particularly when growth is not accompanied by green technological innovation. Conversely, REC is widely regarded as a pathway to decoupling growth from environmental harm. In the European Union, labor and capital investments in renewable energy facilitate both emissions reductions and economic performance.^32^

Numerous studies corroborate this mitigating effect of renewables. REC significantly reduces emissions in EU-15 countries.^33^ A negative link between REC and emissions is also reported for 42 advanced economies using generalized method of moments (GMM) and pooled mean group (PMG) estimators.^34^ Similar conclusions are reached in developing contexts,^35^ while REC adoption is shown to have a stronger mitigating effect in specific regional clusters, including South and Central America and Eurasia.^36^^,^^37^ These results highlight the global relevance of REC policies, albeit with varying effectiveness depending on regional and institutional conditions.

#### Trade, FDI, and oil price fluctuations

Researchers have recently widened their lens. Instead of focusing only on growth and energy use, they now look at variables such as trade openness, FDI, and oil price fluctuations when studying emissions. The results, however, do not all point in the same direction. For example, a positive link between trade and emissions is found in Asia-Pacific countries.^38^ By contrast, negative links are reported for Pakistan and the EU.^33^^,^^39^ These differences tell us that local factors matter a lot. The way a country is structured economically and institutionally can completely change how these variables interact. Ignoring that could lead to models that miss what’s really going on.

Oil prices do not affect CO_2_ emissions uniformly across contexts. A negative association is reported in Gulf economies, potentially reflecting demand-side adjustments to higher prices.^40^ Evidence for African countries suggests different and context-dependent responses. Asymmetric effects between oil exporters and importers are also documented, implying that structural conditions and trade position shape the transmission of oil-price shocks.^41^ Overall, these results support using context-specific modeling rather than a single specification across all settings.

#### Monetary conditions and financial development

Monetary factors including inflation, interest rates, and exchange rates have also been investigated for their indirect effects on CO_2_ emissions. A weak inverse relationship between inflation and emissions is found in a global panel of 189 countries.^42^ Overall, the existing evidence suggests that the impact of monetary conditions on emissions is context-dependent and mediated by structural and institutional factors.

The role of monetary policy, particularly interest rate changes, has been explored in a growing number of studies. Expansionary policies, characterized by low interest rates, often stimulate economic activity and energy demand, thereby increasing emissions. In contrast, contractionary measures can dampen environmental degradation. Evidence from diverse contexts, including Turkey, Brazil, Russia, India, China, and South Africa (BRICS) economies, Asian countries, and G7 nations supports the view that central banks can indirectly influence environmental trajectories through monetary tools.^43^^,^^44^^,^^45^^,^^46^

The role of energy in driving economic growth is well established. Yet, its contribution is not always positive. When energy systems operate inefficiently or without proper oversight, they may actually impede rather than stimulate economic performance, particularly in countries where regulatory capacity is weak.^47^ Additionally, although higher income levels can offer greater protection against environmental risks, they are frequently associated with rising emissions, posing difficult trade-offs for those seeking truly sustainable development.

The relationship between financial development and environmental performance has also drawn attention. Well-developed financial markets and institutions are found to significantly boost REC in the United States over four decades, particularly through enhanced accessibility and market depth.^48^ This suggests that sustainable finance can play a catalytic role in green transitions.

#### Environmental Kuznets curve and cross-country heterogeneity

The EKC remains a widely used theoretical model to assess the relationship between economic growth and environmental degradation. According to the EKC hypothesis, environmental harm initially worsens with growth but eventually declines after a certain income threshold.^49^ Evidence from West Africa suggests that signs of environmental stress may appear early in the development process, well before substantial income gains are realized, consistent with the initial upward phase predicted by the EKC. Using data from 87 middle-income countries, an inverted-U relationship between income and CO_2_ emissions is documented, and the PHH is revisited to suggest that pollution-intensive activities may relocate toward countries with looser environmental regulations.^50^ However, a more complex, N-shaped EKC—where emissions rise, fall, and then increase again after a second income threshold—has been proposed in recent work.^51^^,^^52^ Such growth trajectories are often observed in economies where development remains uneven and fiscal priorities are skewed toward defense rather than social investment; in these contexts, the ecological gains typically associated with rising income levels tend to remain elusive.

Empirical evidence from various European countries reveals that the link between economic growth and environmental impact is far from uniform. Notable disparities in how emissions respond to economic expansion are documented across EU member states.^28^^,^^53^ Evidence also suggests that only seven of the nineteen European countries examined display a long-term connection between economic growth, energy consumption, and emissions.^54^ Rather than supporting a one-size-fits-all approach, these results underscore the need for policy frameworks that reflect the specific economic and institutional realities of each nation.

#### Policy interventions and sectoral transitions

Recent policy evaluations suggest that environmental outcomes from government-led initiatives vary widely across national settings. In China, the drop in industrial CO_2_ emissions has been associated with the Low-Carbon City Pilot scheme, which encouraged firms to innovate and use energy more efficiently.^52^ However, such improvements appear to depend on specific features, including company scale and local conditions. In Pakistan’s agricultural transition, the environmental effects of modernization vary with the type of input: Mechanization and aquaculture tend to raise emissions, whereas soil enhancement and improved seed adoption promote ecological resilience and efficient land use.^55^

In parallel with these macroeconomic and policy-based approaches, an emerging strand of research uses machine-learning techniques to improve the estimation and forecasting of carbon emissions. A recent systematic review highlights that ML models such as neural networks, support-vector machines and ensemble learning methods can substantially enhance predictive accuracy, although challenges related to data quality, computational complexity and model interpretability persist.^8^

#### Synthesis, research gap, and contribution of machine learning

Table 1 summarizes the main empirical studies reviewed above, reporting their study periods, geographical coverage, methodological approaches, and key findings. Taken together, the empirical literature shows that CO_2_ emissions are shaped by a wide range of macroeconomic, energy, trade, financial and policy factors, with substantial heterogeneity acrosscountries and regions. However, three main limitations emerge.

First, many studies rely on restricted sets of variables (typically GDP, total energy use, and a few trade indicators), which makes it difficult to capture the joint influence of demographic, sectoral, financial, and environmental factors on emissions. Second, most contributions use linear or mildly non-linear econometric models (cointegration, autoregressive distributed lag (ARDL), panel regressions) estimated at the country level or on pooled panels, with limited attention to systematic heterogeneity across income groups. This helps explain why the literature reaches conflicting results on the role of trade, oil prices, monetary conditions, or the shape of the EKC, but these inconsistencies are rarely analyzed through models that explicitly allow for complex interactions and non-linear responses. Third, although some recent papers start to apply artificial intelligence and machine learning techniques to CO₂ forecasting, they generally focus on single countries or global aggregates, on methodological development, or on sector-specific applications, and do not systematically assess how the relative importance of macroeconomic predictors and associated factors differs across income categories.

This study addresses these gaps in three ways. (1) It constructs a broad macroeconomic-demographic-energy-trade-environmental dataset that goes beyond the traditional GDP-CO_2_ focus. (2) It explicitly segments the analysis into four World Bank income groups, thereby aligning the empirical design with the structural heterogeneity emphasized in prior work. (3) It employs machine learning methods, in particular RF feature importance and correlation analysis combined with deep learning forecasting models, to uncover non-linear patterns, variable interactions and heterogeneous rankings of predictors that standard econometric approaches are not well equipped to detect. In this sense, machine learning is not only used as a predictive tool, but as a way to generate complementary insights on the hierarchy and behavior of key variables associated with CO_2_ emissions across income groups, while remaining strictly within an associative (non-causal) framework that complements and extends established empirical strategies.

## Results

### Model evaluation

To evaluate the classification models, four standard metrics are reported: precision, recall, F1-score, and the area under the receiver operating characteristic (ROC) curve, i.e. the area under the curve (AUC).^56^ Together, these indicators provide a complementary assessment of classification performance and class separability across decision thresholds.

• **Precision** quantifies the proportion of correctly predicted positive instances among all instances classified as positive. It reflects the model’s ability to avoid false positives:

(Equation 1) $$Precision=\frac{TP}{TP\;+\;FP}$$

• **Recall**, also known as sensitivity, measures the proportion of actual positive cases that were correctly identified by the model. It is especially critical when the cost of false negatives is high:

(Equation 2) $$Recall=\frac{TP}{TP\;+\;FN}$$

• **F1-score** represents the harmonic mean of precision and recall, offering a balanced metric that is particularly informative when class distributions are imbalanced or when both false positives and false negatives are of concern:

(Equation 3) $$F1-score=2\;x\;\frac{Precision\;x\;Recall}{Precision\;+\;Recall}$$

• **AUC-ROC** evaluates the model’s ability to distinguish between classes across a range of threshold values. An AUC-ROC score close to 1 indicates strong discriminatory ability, whereas a value below 0.5 suggests that the model performs worse than random classification.

These metrics capture complementary aspects of classification performance. Precision reflects the reliability of positive predictions, recall measures the ability to identify positive cases, and the F1-score balances both. The AUC-ROC summarizes discrimination ability across decision thresholds, providing a threshold-independent view of model separability. Interpreting these indicators jointly yields a more robust assessment of model behavior.

### Algorithms’ accuracy and performance

Using World Bank WDI data (1990–2023), Table 2 reports 10-fold cross-validated performance for gradient boosting (GB) and RF across four income groups using AUC, F1-score, precision, and recall.

**Table 1:** The summary of the literature review

Study	Period	Region/Countries	Method	Key findings
Bilan et al.^32^	1995–2015	OECD countries	Panel econometric analysis (cointegration/ARDL-type framework)	Renewable energy consumption reduces CO_2_ emissions, while economic growth increases emissions
Dogan et al.^33^	1990–2012	Top renewable-energy-consuming countries	Panel cointegration and Dumitrescu-Hurlin causality	Growth and energy use increase emissions, trade openness reduces emissions in the long run
Ito et al.^34^	2002–2011	42 developing countries	Difference GMM and PMG-ARDL	Renewable energy reduces emissions and supports long-run economic growth
Bhattacharya et al.^35^	1991–2012	38 top renewable-energy-consuming countries	Panel cointegration (PMG, MG, DFE estimators)	Renewable energy consumption promotes long-run economic growth
Rahman et al.^38^	1960–2016	17 Asia-Pacific countries	Panel estimation	Energy use, economic growth, financial development, and trade increase CO_2_ emissions; squared GDP reduces emissions (EKC validated)
Mahmood et al.^40^	1980–2019	GCC countries	Nonlinear ARDL (NARDL) with asymmetrical cointegration	Oil price shocks have asymmetric and country-specific effects on CO_2_ emissions.
Pata et al.^48^	1980–2019	USA	Fourier wavelet quantile causality test	Financial development—especially the depth and access of financial markets—promotes renewable energy consumption in the medium and long run.
Naqvi et al.^50^	1990–2017	87 middle-income countries	Panel models	EKC confirmed; FDI, urbanization and natural resource use increase ecological footprint (PHH supported).
Yang et al.^52^	2007–2014	China—industrial firms in low-carbon pilot cities	Time-varying difference-in-differences (DID) with firm-level matched data (PSM-DID robustness)	Low-carbon city pilot (LCCP) intervention significantly reduces firms’ electricity consumption intensity, with heterogeneous effects across ownership and regions.
Al Nuaimi et al.^8^	–	Global	Systematic literature review of ML-based CO_2_ estimation	ML techniques (ANN, SVM, ensemble and hybrid models) improve accuracy of carbon-emission estimation; strongest evidence in transport sector; gaps remain for energy and industry

**Table 2:** Cross-validated performance of GB and RF models by income group

Income group	Model	AUC	F1-score	Precision	Recall
Low-income	GB	0.960	0.845	0.846	0.845
	RF	**0.976**	**0.880**	**0.881**	**0.879**
Lower-middle-income	GB	0.979	0.936	0.940	0.935
	RF	**0.991**	**0.938**	**0.941**	**0.938**
Upper-middle-income	GB	0.986	0.928	0.928	0.928
	RF	**0.992**	**0.957**	**0.956**	**0.957**
High-income	GB	0.996	0.963	0.963	0.963
	RF	**0.998**	**0.972**	**0.973**	**0.972**

Authors’ calculations based on World Bank World Development Indicators (WDI), 1990–2023; performance metrics computed using 10-fold cross-validation.

Random Forest consistently outperforms Gradient Boosting across all income groups, as indicated by higher AUC and F1-score values in each income group.

Bold values indicate the best cross-validated performance obtained for each income group (Random Forest).

Table 2 indicates that RF consistently achieves higher classification performance than GB across income groups. The gap is modest in low- and lower-middle-income countries, but becomes more substantial in upper-middle- and high-income groups, where RF approaches near-ceiling discrimination (AUC ≈0.99–1.00). Therefore, RF is selected as the primary model for the feature-importance analysis in the subsection “Feature importance of macroeconomic variables using the random forest algorithm”, while GB is retained as a benchmark for comparison.

### Feature importance of macroeconomic variables using the random forest algorithm

RF feature importance is used to quantify the relative contribution of each predictor to CO_2_ emissions within each income group. Using World Bank WDI data (1990–2023), Table 3 reports the resulting importance scores computed from the final RF models estimated separately for low-, lower-middle-, upper-middle-, and high-income countries.

**Table 3:** Random forest feature importance by income group

Variables	Low-income	Lower-middle-income	Upper-middle-income	High-income
GDP Growth (per capita)	1.124	12.387	4.285	0.435
Agriculture (VA)	1.539	1.342	1.921	4.603
Industry (VA)	3.054	5.837	22.336	12.365
Services (VA)	0.991	1.423	3.333	3.559
Population Growth	2.941	4.653	27.965	4.110
Forest Area	14.142	7.072	2.319	5.348
Exports	1.693	2.749	1.295	7.200
Imports	4.746	24.268	**31.121**	**52.327**
FDI	4.986	1.821	0.803	0.526
Renewable Energy	**64.135**	9.972	3.651	7.338
Fuel Imports	0.644	**28.471**	0.964	2.182

Authors’ calculations based on World Bank World Development Indicators (WDI), 1990–2023; random forest feature importance computed from the final RF models estimated separately for each income group.

The highest feature-importance score in each income group is highlighted in bold, indicating the dominant predictors of CO_2_ emissions.

In low-income countries, REC is the dominant driver (64.14%), indicating that the energy mix plays a central role in emissions dynamics (Table 3). Forest area is the second most influential factor (14.14%), highlighting sensitivity to land use and deforestation. External engagement (FDI: 4.99%; imports: 4.75%) and structural pressures (industry value added: 3.05%; population growth: 2.94%) contribute moderately, while the remaining variables have comparatively limited importance.

In lower-middle-income countries, CO_2_ emissions are primarily driven by fuel imports (28.47%) and imports of goods and services (24.27%), indicating a strong reliance on externally sourced energy and carbon-intensive trade (Table 3). GDP per capita growth is the third most influential factor (12.39%), suggesting that economic expansion remains closely associated with rising emissions. Industry value added (5.84%) and population growth (4.65%) contribute moderately, capturing structural and demographic pressures on energy demand. Environmental variables also retain a non-negligible role, with renewable energy (9.97%) and forest area (7.07%) showing moderate importance, while the remaining predictors have comparatively limited influence.

In upper-middle-income countries, imports are the dominant predictor of CO₂ emissions (31.12%), followed by population growth (27.97%) and industry value added (22.34%), indicating that trade exposure, demographic pressure, and industrial activity jointly shape emission patterns in this group (Table 3). Services (3.33%) and REC (3.65%) show moderate importance, suggesting that low-carbon transitions remain present but not yet strong enough to offset industrial pressures. GDP per capita growth (4.29%) also contributes, indicating that ongoing development and modernization may still translate into higher energy demand when efficiency gains and clean technologies are insufficient. The remaining variables have comparatively limited influence.

In high-income countries, imports are the strongest predictor of CO_2_ emissions (52.33%), consistent with carbon-intensive consumption patterns and the outsourcing of emissions through global supply chains (Table 3). Industry value added (12.37%) and exports (7.20%) remain influential, indicating that advanced industrial activity and trade continue to contribute to emissions despite technological sophistication. Renewable energy consumption shows moderate importance (7.34%), while services (3.56%) and population growth (4.11%) contribute to a lesser extent. GDP per capita growth (0.44%) and FDI (0.53%) have minimal importance, suggesting that in these economies, aggregate economic expansion and foreign capital inflows are not primary predictors of emissions in the model.

Using World Bank WDI data (1990–2023), Table 4 shows that in low-income countries, CO_2_ emissions are moderately positively correlated with industry value added (0.288) and exports (0.244), consistent with early-stage industrialization and trade expansion contributing to emissions. In contrast, REC exhibits a strong negative correlation (−0.694), indicating that greater renewable adoption is associated with lower emissions. Other variables display weak correlations in magnitude, suggesting limited direct linear association with CO_2_ emissions in this group.

In lower-middle-income countries, Table 4 indicates that CO_2_ emissions are most positively correlated with fuel imports (0.279), consistent with greater reliance on imported fossil fuels being associated with higher emissions. GDP per capita growth (0.081) and industry value added (0.059) show only weak positive correlations. In contrast, imports (−0.168), REC (−0.122), and forest area (−0.107) exhibit modest negative correlations, while the remaining variables display weak correlations in magnitude, suggesting limited direct linear association with emissions in this group.

**Table 4:** Correlation coefficients between CO_2_ emissions and macroeconomic variables across income groups

Variables	Low-income	Lower-middle-income	Upper-middle-income	High-income
GDP Growth (per capita)	−0.039	0.081	0.07	−0.009
Agriculture (VA)	−0.226	−0.022	−0.012	−0.07
Industry (VA)	0.288	0.059	0.153	0.014
Services (VA)	0.055	−0.049	−0.071	0.061
Population growth	−0.159	−0.072	−0.04	−0.039
Forest Area	0.042	−0.107	−0.097	0.041
Exports	0.244	−0.084	−0.124	−0.241
Imports	−0.006	−0.168	**−0.191**	**−0.259**
FDI	−0.041	−0.054	−0.045	−0.047
Renewable energy	**−0.694**	−0.122	−0.044	−0.074
Fuel imports	−0.001	**0.279**	−0.001	−0.017

Authors’ calculations based on World Bank World Development Indicators (WDI), 1990–2023; correlation coefficients computed between CO_2_ emissions and macroeconomic variables by income group.

The highest correlation (in absolute value) in each income group is highlighted in bold to indicate the strongest statistical association with CO_2_ emissions.

In upper-middle-income countries, Table 4 shows that CO_2_ emissions are moderately positively correlated with industry value added (0.153), indicating that industrial activity remains a key contributor to emissions in this group. GDP per capita growth also exhibits a weak positive correlation (0.07), suggesting a limited association between economic expansion and emissions. Renewable energy consumption displays a small negative correlation (−0.044), pointing to early but still modest mitigation effects. Imports are negatively correlated with emissions (−0.191), while the remaining variables show weak correlations in magnitude, indicating limited direct linear influence.

In high-income countries, Table 4 indicates generally weak linear associations between macroeconomic variables and CO_2_ emissions. The most notable correlations are negative for imports (−0.259) and exports (−0.241), consistent with trade-related shifts in the location of emissions along global value chains. Renewable energy consumption is also negatively correlated with emissions (−0.074), while GDP per capita growth shows a near-zero correlation (−0.009). The remaining variables exhibit correlations of small magnitude, suggesting limited direct linear influence in this group.

The main findings of this study can be synthesized as follows.

• **Model performance:** RF consistently achieves stronger cross-validated classification performance than GB across income groups, motivating its use for subsequent interpretation analyses (Table 2).
• **Driver heterogeneity:** feature-importance rankings indicate a clear shift in the most important predictors with development level, from energy-mix and natural-capital factors in low-income countries toward trade- and industry-related factors in upper-middle- and high-income groups (Table 3).
• **Correlation patterns:** simple correlations broadly support this heterogeneity, with stronger energy-related associations in low-income countries and weaker—sometimes inverted—linear relationships in high-income economies (Table 4).

Overall, these results highlight that the key predictors of CO_2_ emissions are highly income-dependent, supporting differentiated interpretation across development stages.

**Figure 1:**
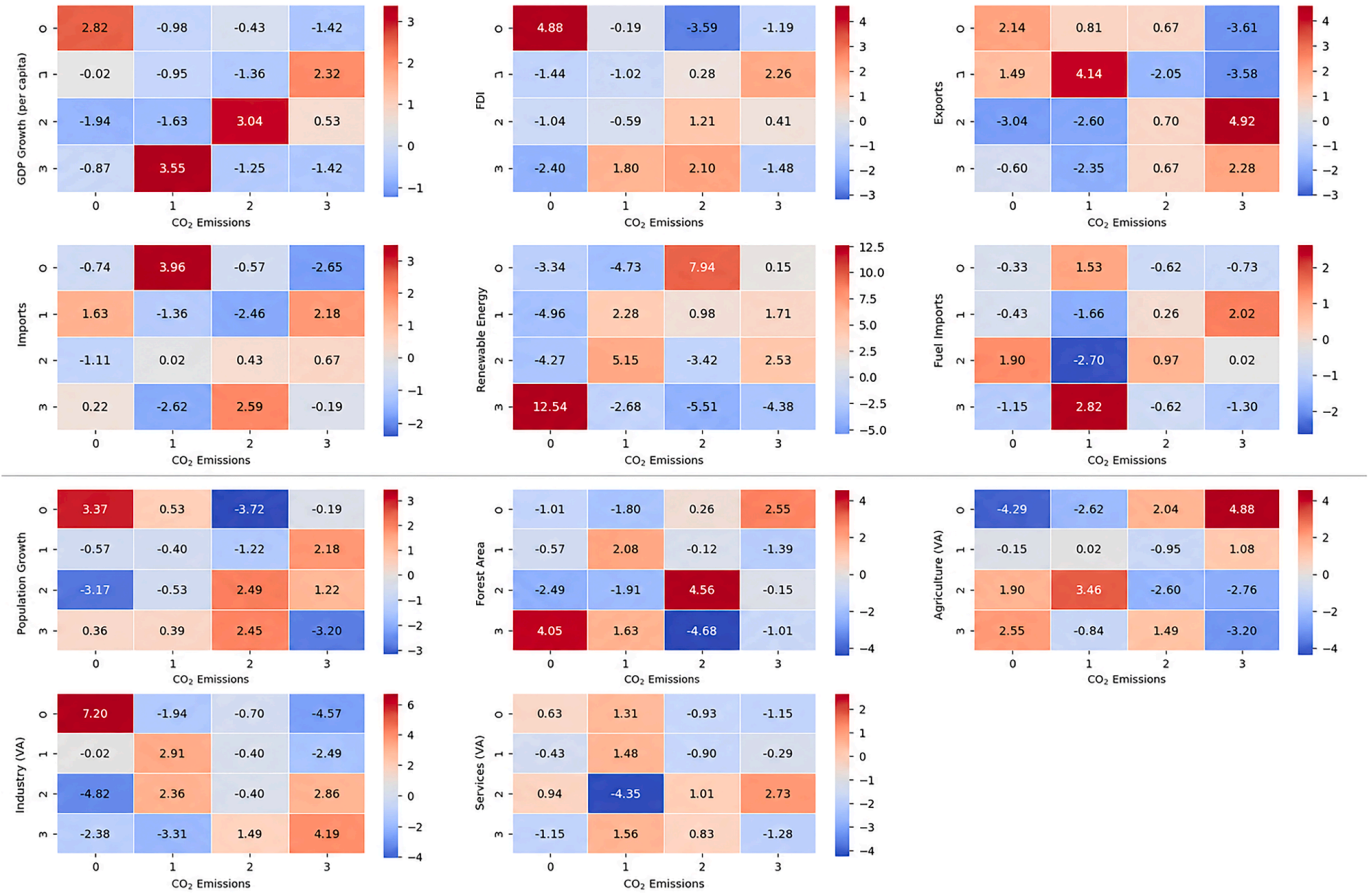
Sieve diagram for low-income countries

**Figure 2:**
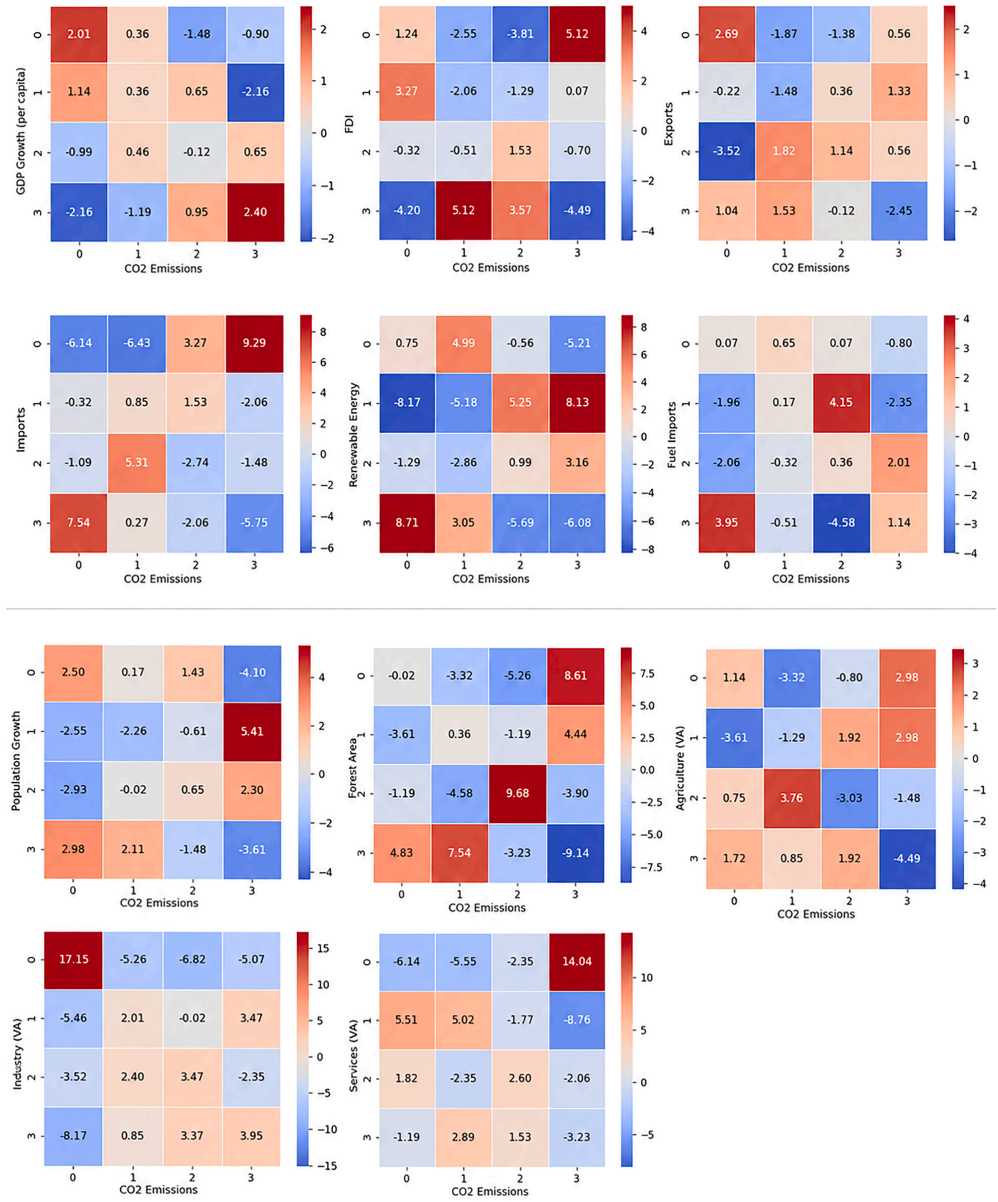
Sieve diagram for lower-middle-income countries

**Figure 3:**
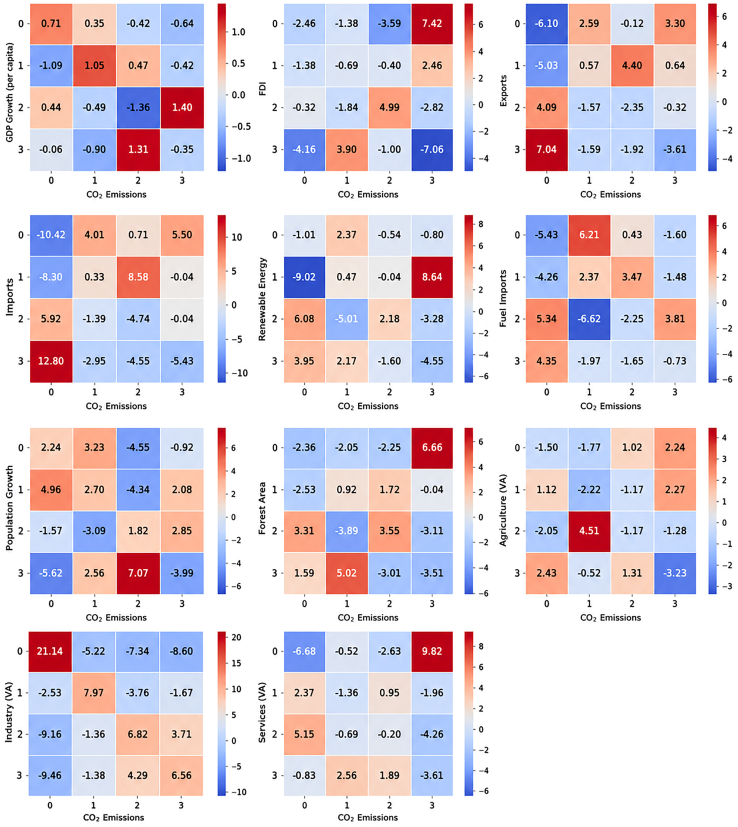
Sieve diagram for upper-middle-income countries

**Figure 4:**
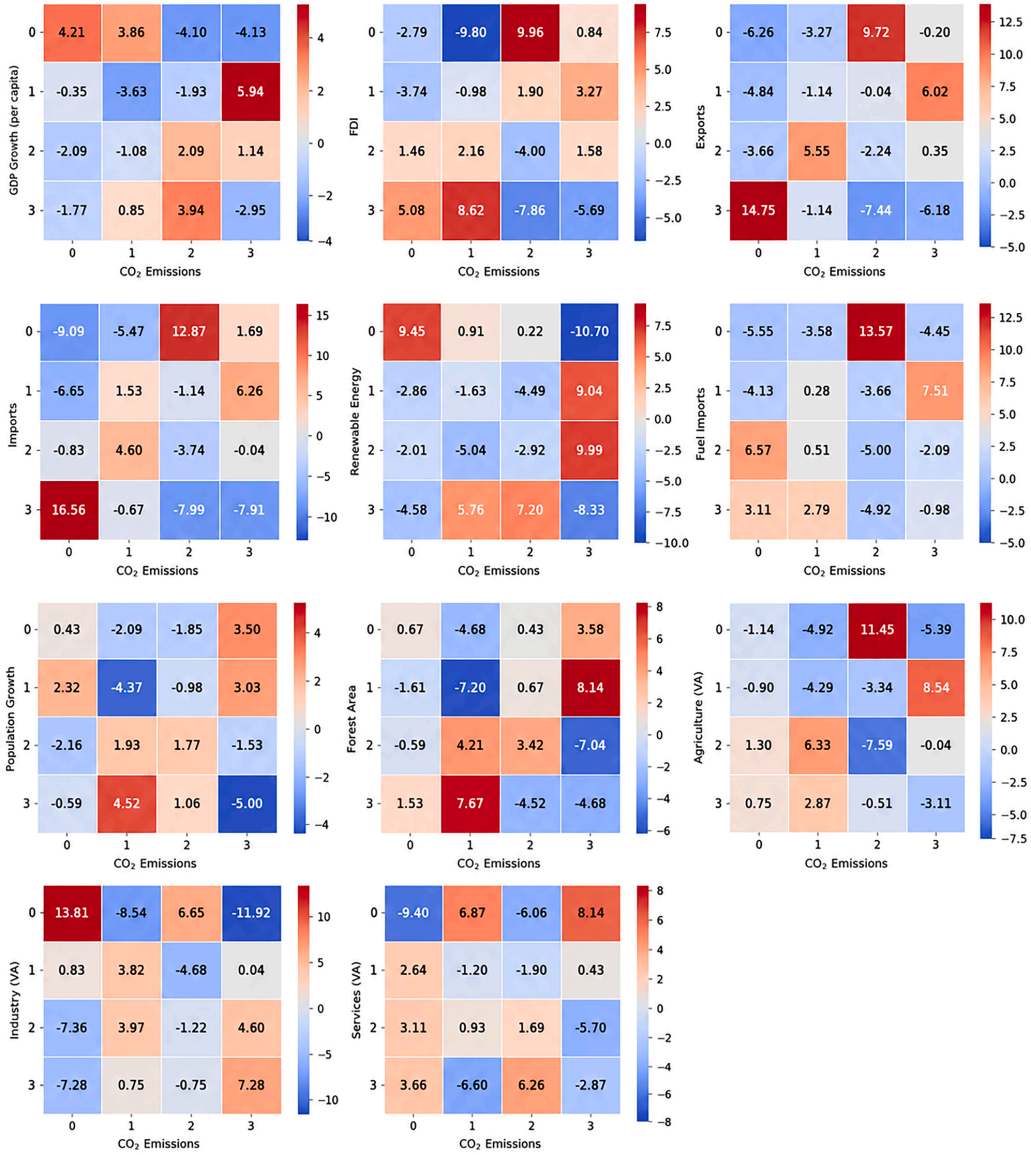
Sieve diagram for high-income countries

Figures 1, 2, 3, and 4 offer a practical illustration of how variables connect, both in strength and direction, through color. For example, deeper red tones signal strong positive links, while darker blue tones reflect strong negative ones. Paler colors suggest that the relationship is weaker or neutral. These visual tools help unpack how economic indicators and CO_2_ emissions interact across various income groups, making the patterns clearer and easier to compare.

### Deep learning-based modeling

#### Hyperparameter tuning and experimental setup

To ensure optimal model performance, an extensive hyperparameter search was conducted. Specifically, the number of time steps was varied across 5, 10, 15, and 20 to investigate the effect of sequence length on the model’s ability to capture temporal dependencies. Increasing the number of time steps enables the model to learn from longer historical patterns but also introduces higher complexity and potential risks of overfitting.

Different hidden-layer sizes (32, 64, 128, 256, and 512) were evaluated to examine how network behavior changes as model complexity increases. As expected, adding more units gave the model more flexibility to learn from the data. But with that came challenges too: training became heavier, and without proper constraints, the model started picking up noise instead of general patterns.

Several batch sizes (16, 32, and 64) were tested to assess their effect on training dynamics. When using smaller batches, the model updated more often, which sometimes led to faster learning but also made the training process less stable. On the other hand, with larger batches, updates were more consistent, though training became slower and demanded more memory.

Regarding optimization, two optimizers were compared in the experimental setup: Adam and RMSprop. Adam adapts the learning rate as training progresses, which helps it speed up convergence in many cases. RMSprop works differently—it’s often used with sequential data because it modifies the learning rate depending on the recent behavior of the gradients.

The same search space was explored for each of the three deep-learning models (Multilayer perceptron (MLP), recurrent neural network (RNN), and long short-term memory (LSTM)) as a full grid search over all combinations of the values reported in Table 5. This grid comprises 4 × 5 × 3 × 2 = 120 candidate configurations per model, each of which was trained and evaluated. The configuration minimizing the validation loss was selected for each model, with early stopping on the validation set used to limit overfitting; the resulting optimal configurations and their test losses are reported in Tables 6, 7, and 8. The remaining training hyperparameters that were not part of the grid, namely the learning rate, the maximum number of training epochs, and the early-stopping patience, were kept at the default values of the implementation and held constant across all models and configurations, so that performance differences are attributable to the searched hyperparameters rather than to differences in the training setup.

**Table 5:** Hyperparameter search space and optimal configurations for the deep-learning models

Hyperparameter	Search range	MLP (optimal)	RNN (optimal)	LSTM (optimal)
Time steps (sequence length)	{5, 10, 15, 20}	**15**	**5**	**5**
Hidden units	{32, 64, 128, 256, 512}	**32**	**512**	**512**
Batch size	{16, 32, 64}	**64**	**16**	**64**
Optimizer	{Adam, RMSprop}	**Adam**	**Adam**	**RMSprop**

Authors’ calculations based on World Bank WDI (1990–2023); hyperparameter search space and optimal configurations obtained by full grid search for the MLP, RNN, and LSTM models.

The same grid was applied to all three models, giving 4 × 5 × 3 × 2 = 120 candidate configurations per model. The optimal configuration for each model corresponds to the lowest validation loss.

Bold values indicate the optimal configuration selected for each model.

**Table 6:** Optimal hyperparameters and test loss achieved by the MLP

Model	Multilayer Perceptron (MLP)			
Hyperparameters	Time steps	Units	Batch size	Optimizer
	15	32	64	Adam
Test loss	0.00709			

Authors’ calculations based on World Bank WDI (1990–2023); hyperparameter tuning results for the MLP model.

**Table 7:** Optimal hyperparameters and test loss achieved by the RNN

Model	Recurrent neural network (RNN)			
Hyperparameters	Time steps	Units	Batch size	Optimizer
	5	512	16	Adam
Test loss	0.00827			

Authors’ calculations based on World Bank WDI (1990–2023); hyperparameter tuning results for the RNN model.

**Table 8:** Optimal hyperparameters and test loss achieved by the LSTM

Model	Long short-term memory (LSTM)			
Hyperparameters	Time steps	Units	Batch size	Optimizer
	5	512	64	RMSprop
Test loss	0.00785			

Authors’ calculations based on World Bank WDI (1990–2023); hyperparameter tuning results for the LSTM model.

During the hyperparameter tuning process, particular care was given to adjusting the model’s architecture and training setup to achieve an effective balance between predictive accuracy, computational efficiency, and resilience to variability. To assess and improve model performance, the dataset was divided into three distinct subsets: 80% for training, 15% for validation, and 5% for testing. The training set helped the model learn, and the validation data were used to tweak the hyperparameters and limit overfitting, especially through early stopping. Importantly, this 80/15/5 partition is performed along the time axis rather than by random shuffling: Within each country, the earliest years are assigned to training, the following years to validation, and the most recent years to testing, so that the test set is strictly posterior to the training period and no future observation is used to predict a past one. The sliding-window sequences underlying the recurrent models (the time steps of 5–20 years reported above) are likewise constructed within each country in chronological order, with the train/validation/test boundaries placed between dates, so that no individual sequence straddles the split and no temporal overlap occurs across subsets. This design ensures that both the cross-validation procedure and the 80/15/5 split respect the temporal dependence of the data. The final model was then evaluated on a fully held-out test set to provide an unbiased estimate of generalization performance. This design enabled the use of most observations for training while preserving the reliability of validation and testing.

#### Deep learning models: MLP, RNN, and LSTM

##### Multilayer perceptron (MLP)

Using World Bank WDI data (1990–2023), Table 6 shows the optimal hyperparameter configuration and the corresponding performance achieved by the MLP model. Using the Adam optimizer, the architecture, with 15-time steps, 32 units, and a batch size of 64, achieved a test loss of 0.00709.

**Figure 5:**
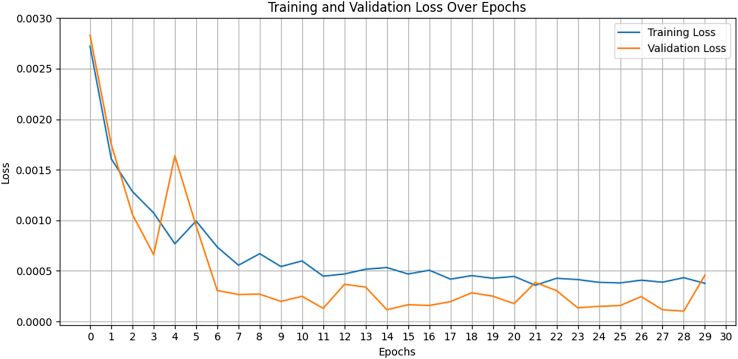
Training and validation loss curves over epochs for the MLP Model

Figure 5 shows a progressive decline in both training and validation loss curves as the model learns over time. The close correspondence between the two curves indicates a stable learning process, with no major signs of overfitting. This consistency suggests that the MLP captures the structure of the data effectively while maintaining its capacity to generalize across previously unobserved samples.

During the initial epochs, a noticeable drop in both training and validation losses suggests that the model is effectively learning core patterns from the dataset. As training proceeds, the curves begin to stabilize, reflecting a phase of convergence and reliability. This trend reflects the model’s consistent behavior and its capacity to maintain learning efficiency under stable conditions.

Based on these results, the MLP model configured with the selected hyperparameters exhibits a stable learning dynamic and satisfactory generalization capabilities. To deepen the analysis, further evaluation will involve comparing these findings with those from recurrent-based models, in order to provide a comprehensive assessment of the deep learning models are discussed in the following.

##### Recurrent neural network (RNN)

Using World Bank WDI data (1990–2023), Table 7 presents the optimal hyperparameter settings and performance metrics for the RNN model. The configuration, composed of 5 time steps, 512 units, a batch size of 16, and the Adam optimizer, resulted in a test loss of 0.00827.

**Figure 6:**
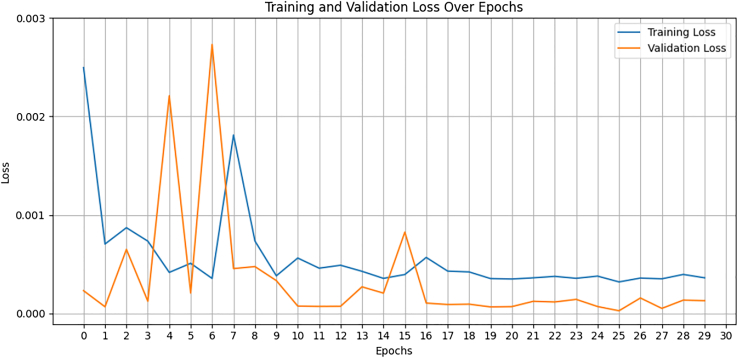
Training and validation loss curves over epochs for the RNN Model

As shown in Figure 6, the training and validation loss curves reveal greater variability than those of the MLP model. While the training loss gradually decreases over epochs, the validation curve shows noticeable instability at the beginning of training. This suggests that the model may have difficulty consistently capturing and generalizing temporal patterns, particularly in sequential dependencies present in the data.

Despite the eventual reduction of both losses, the instability observed in the validation curve indicates potential difficulties in generalization, with the model being sensitive to variations across batches. Such behavior is characteristic of simple RNN architectures, which are known to struggle with learning long-range dependencies due to issues such as vanishing gradients.

Overall, the RNN model demonstrates a relatively less stable learning process compared to expectations for sequence modeling tasks. Further evaluation with advanced recurrent structures, such as LSTM networks, is conducted to assess whether such challenges can be mitigated and to determine the most suitable architecture for the task at hand.

##### Long short-term memory (LSTM)

Using World Bank WDI data (1990–2023), Table 8 details the optimal hyperparameter configuration and the associated performance metrics obtained by the LSTM model. The architecture, incorporating 5 time steps, 512 units, a batch size of 64, and optimized with RMSprop, achieved a test loss of 0.00785.

**Figure 7:**
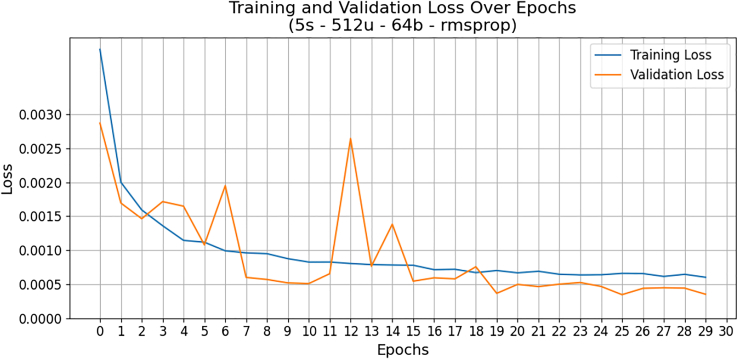
Training and validation loss curves over epochs for the LSTM Model

The training and validation loss curves, depicted in Figure 7, indicate a relatively stable learning process with a gradual reduction of both losses across epochs. Compared to the RNN model, the LSTM demonstrates improved stability, with fewer pronounced fluctuations in the validation loss, particularly in the later stages of training. This behavior reflects the LSTM’s ability to better handle sequential dependencies and mitigate issues such as vanishing gradients, which often affect simpler recurrent architectures.

Nevertheless, some sporadic spikes in the validation loss are observed, suggesting occasional variability in the model’s generalization performance. These variations may stem from the inherent complexity of the dataset or minor instabilities during training despite the LSTM’s enhanced memory capabilities.

Overall, the LSTM model exhibits a more robust and consistent training dynamic compared to the standard RNN, while still maintaining a moderate level of variability in validation performance. A comparative analysis with the other models will be conducted subsequently to assess the overall suitability of each architecture for the modeling task.

Following the evaluation of the MLP, RNN, and LSTM models, a comparative analysis was conducted based on their optimal hyperparameter configurations and corresponding performance metrics.

As shown in Tables 6, 7, and 8, the MLP model achieved the lowest test loss (0.00709), outperforming both the LSTM (0.00785) and the RNN (0.00827) models. This suggests that, in the context of this study, the predictive task does not heavily rely on sequential dependencies, thereby favoring the MLP’s ability to capture complex patterns without the need for recurrent structures.

From an economic perspective, this pattern indicates that most of the variability in the emissions indicator is explained by structural differences in macroeconomic conditions (such as trade structure, energy mix, and industrial composition) rather than by high-frequency temporal shocks. In other words, once the cross-sectional configuration of macroeconomic variables is taken into account, additional sequential information brings limited predictive gains. This finding is consistent with the slow-moving nature of key factors such as capital accumulation, demographic trends, and energy infrastructure, which tend to evolve gradually over time rather than exhibiting abrupt short-term reversals.

The training and validation loss curves (Figures 5, 6, and 7) further support these findings. The MLP model (Figure 5) exhibits a smooth and consistent convergence of both training and validation loss, with no significant overfitting observed. This indicates a stable learning process and strong generalization capabilities. Conversely, the RNN model (Figure 6) demonstrates noticeable fluctuations in validation loss, particularly during the early epochs. This instability suggests difficulties in learning long-range dependencies effectively, possibly due to vanishing gradient issues inherent to simple RNN architectures. The LSTM model (Figure 7), although more stable than the RNN, displays sporadic spikes in validation loss, indicating occasional generalization challenges despite its architectural improvements in handling sequential data.

Overall, while RNN and LSTM architectures are generally more suitable for sequential modeling tasks, the MLP model demonstrated superior effectiveness for the dataset considered in this study. This performance is likely attributable to the limited presence of strong temporal dependencies among the input features. Consequently, the MLP architecture emerges as the most appropriate choice for the problem under investigation, striking a balance between training stability, low test error, and robust generalization capabilities.

The forecasting exercise was conducted using a MLP neural network architecture, based on historical data extending up to 2023. The dataset was divided into training (80%), validation (15%), and testing (5%) subsets to ensure rigorous model development and evaluation. The MLP model was selected for its ability to capture complex, nonlinear relationships among macroeconomic and environmental variables, providing robust predictive capabilities. Because the models are trained on observations up to 2023 and then used to project the subsequent year (2024), this forecasting exercise is intrinsically out-of-time and provides a direct, leakage-free assessment of the models’ ability to generalize to future periods.

The predictions for 2024 reveal significant disparities across country income groups. High-income countries are projected to experience a growth of +45.7% in the targeted indicator, reflecting the sustained expansion of economic activities and associated consumption patterns. In low-income countries, the forecast indicates a particularly sharp increase of +710.1%, suggesting accelerated industrialization and urbanization processes that could intensify environmental pressures if unaccompanied by mitigation measures.

In contrast, upper-middle-income countries are expected to witness a contraction of −29.2%, potentially indicative of successful decarbonization efforts or structural economic transformations. Lower-middle-income countries are projected to register a more moderate decline of −16.7%, reflecting a gradual transition toward more sustainable development pathways.

These forecasts are consistent with the heterogeneity in macroeconomic patterns highlighted by the RF analysis. In low-income countries, where renewable energy use remains limited and forest resources are under pressure, the projected surge in emissions reflects the combination of rapid industrialization and weak environmental safeguards. In contrast, the expected declines in upper-middle- and lower-middle-income countries are aligned with their gradual structural reorientation toward less carbon-intensive activities and, in some cases, stronger environmental and trade regulations. For high-income countries, the projected increase is compatible with the strong influence of imports and industrial output identified in the feature importance ranking, suggesting that part of their carbon footprint continues to be shaped by consumption-based emissions embedded in global value chains.

These heterogeneous patterns underline the differentiated trajectories across income groups and reinforce the necessity for tailored strategies to address the evolving environmental and economic challenges specific to each context.

## Discussion

### Discussion in the context of prior literature

First, the strong influence of REC in reducing emissions in low-income countries is consistent with prior work showing that renewable deployment contributes to mitigation outcomes across diverse regions.^57^^,^^58^^,^^59^ These results align with studies that document a negative association between renewable energy use and emissions in both advanced and developing economies, reinforcing the argument that clean-energy expansion is a key pillar of decarbonization strategies in resource-constrained settings.

Second, the positive association between economic growth, industrial activity, and emissions in lower-middle- and upper-middle-income countries echoes the EKC framework, whereby emissions tend to increase during industrialization before declining at higher income levels. Our findings also support earlier evidence that structural transformation toward manufacturing and carbon-intensive energy systems remains a key driver of environmental degradation during intermediate stages of development.^60^^,^^61^^,^^62^ In emerging economies such as Mexico, for example, ARDL and wavelet-coherence evidence confirms that trade openness, energy use, capital formation, and real growth exert positive long-run effects on CO_2_ emissions while the EKC hypothesis holds.^62^ At the same time, the moderate mitigating role of renewable energy and forest area in these economies suggests the early emergence of partial decoupling effects, thereby extending previous studies that focus primarily on high-income contexts.

Third, the dominant role of imports in shaping emissions in high-income countries is highly consistent with the consumption-based emissions and carbon-leakage literature. This supports prior evidence indicating that environmental pressures are increasingly embodied in global value chains rather than generated domestically.^63^^,^^64^^,^^65^ Our results therefore reinforce the argument that mitigation policies in advanced economies must explicitly account for the carbon intensity of imported goods, rather than focusing solely on domestic production processes.

Finally, the generally weak correlation between GDP per capita growth and emissions in high-income economies supports the hypothesis of partial decoupling between output and environmental degradation at advanced development stages. This outcome is aligned with studies showing that, in developed and Organisation for Economic Co-operation and Development (OECD) economies, relative or even strong decoupling between economic growth and CO_2_ emissions is largely driven by technological innovation, energy-efficiency gains, changes in energy intensity and structural shifts in the economy.^66^^,^^67^ However, the continuing influence of industrial and trade-related variables indicates that complete decarbonization remains elusive, thereby extending the existing literature by highlighting the persistence of residual structural patterns linked to higher emission levels.

Taken together, these results position our contribution at the intersection of three strands of research: (1) the emissions-growth nexus, (2) the role of trade and globalization, and (3) the environmental benefits of renewable energy adoption. By showing that the relative importance of these predictors varies systematically across income groups, this study moves beyond single-country or globally aggregated analyses and provides empirical support for tailoring mitigation strategies to countries’ structural development conditions.

### Conclusion

This study applied machine-learning methods, using RF models, to identify the main predictors of CO_2_ emissions across low-, lower-middle-, upper-middle-, and high-income countries. The results reveal substantial heterogeneity in predictive determinants across development stages, indicating that a single one-size-fits-all framework is insufficient to characterize emission patterns. Feature-importance rankings point to a clear transition from energy-mix and natural-capital factors in low-income economies toward trade- and industry-related predictors in upper-middle- and high-income groups, consistent with increasingly globalized production and consumption patterns. Correlation results broadly corroborate this heterogeneity, showing stronger energy-related associations in low-income countries and generally weaker linear links in high-income economies, where emissions may be more strongly associated with technology, regulation, and the structure of global value chains.

Importantly, the observed cross-group differences are consistent with evidence that the mitigation impact of renewable energy depends on countries’ capacity to finance and sustain the energy transition, including through green finance instruments such as green bonds.^68^ They also align with findings emphasizing spatial and structural heterogeneity in the economic and environmental effects of energy consumption, implying that transitions can yield non-uniform outcomes across contexts.^69^ Overall, these findings confirm that emission dynamics are highly context-dependent, supporting differentiated mitigation strategies aligned with countries’ structural characteristics and development constraints.

From a methodological perspective, the study also illustrates how different branches of artificial intelligence can be combined to generate policy-relevant insights on CO_2_ emissions. Deep learning models (MLP, RNN, and LSTM) are used to forecast the future trajectory of an aggregate emissions indicator conditional on macroeconomic conditions, while the RF algorithm provides an interpretable ranking of the macroeconomic factors most strongly associated with emissions across income groups. In this way, the AI-based framework jointly delivers high predictive accuracy and an economically meaningful decomposition of heterogeneity in emission patterns. At the same time, the analysis remains explicitly non-causal: The identified predictors are interpreted as variables that are strongly linked to CO_2_ emissions in a predictive sense rather than as structural causal effects.

### Policy implications and recommendations

Based on the findings of this study, the following targeted policy recommendations are proposed.

• **Low-income countries (renewables and forest area as key predictors):** Scale decentralized renewables (mini-grids/solar home systems where grid access is limited) and implement targeted programs to replace the most carbon-intensive fuels used in power generation and household energy. Strengthen forest monitoring and enforcement (e.g., satellite-based tracking, anti-illegal logging measures, and incentives for re/afforestation), and condition future industrial projects on best-available low-emission technologies.
• **Lower-middle-income countries (fuel imports and imports as main predictors):** Reduce fossil-fuel import dependence through minimum energy-performance standards for industrial equipment and buildings, mandatory energy audits for large emitters, and targeted retrofitting programs. Use public procurement and product standards to shift toward lower-carbon imported inputs (e.g., machinery, vehicles, and industrial intermediates), consistent with evidence that green taxation and supportive trade frameworks can facilitate cleaner transitions.^70^
• **Upper-middle-income countries (industry, population pressures, and trade exposure):** Prioritize industrial decarbonization via sectoral benchmarks (e.g., cement/steel), time-bound adoption of cleaner production technologies, electrification where feasible, and urban measures that curb energy intensity (building codes, mass transit expansion, compact-city planning).
• **High-income countries (imports as dominant driver):** Complement territorial targets with supply-chain measures addressing embodied carbon in trade: mandatory disclosure of product/supply-chain emissions, low-carbon public procurement, and incentives for firms to decarbonize upstream production.
• **Policy design safeguards:** Carbon pricing can complement these measures, but impacts depend on design and revenue recycling; evidence from a general-equilibrium framework highlights the importance of gradual implementation and recycling schemes.^71^ Renewable expansion should be accompanied by systematic environmental impact assessment and mitigation measures to manage localized trade-offs reported in wind-farm assessments.^72^

Overall, these recommendations translate the empirical determinants into implementable policy levers tailored to development stages.

### Limitations of the study

Despite the valuable insights generated by this study, several limitations should be acknowledged to guide future research efforts.

First, the analysis adopts a production-based accounting approach to emissions, associating carbon outputs with the location of production rather than final consumption. Consequently, the significant role of international trade and carbon outsourcing, particularly relevant for high-income countries, may be underestimated. Future studies should integrate consumption-based accounting frameworks to better capture the true environmental footprint associated with globalized supply chains.

Second, while the feature importance and correlation analyses provide robust evidence on current emission predictors across income groups, the study does not account for potential future dynamics. Ongoing global trends such as energy transition efforts, technological innovation, and geopolitical shifts may substantially alter the relationship between macroeconomic indicators and CO_2_ emissions. Thus, the findings reflect the prevailing conditions at the time of analysis and should be interpreted in light of the existing economic and technological context as predictive associations rather than causal effects.

Third, the reliance on aggregated macroeconomic variables, such as total imports, exports, and industrial value added, limits the granularity of the analysis. Emission profiles can vary significantly within these broad categories depending on the composition of goods and services. A more detailed sectoral breakdown would allow for a finer understanding of which subcomponents drive emissions the most.

Fourth, the methodology is based on correlation analysis, which, while informative, does not establish causal relationships between macroeconomic variables and emissions. As such, caution must be exercised when inferring direct causal links from the observed associations.

Finally, the study does not fully differentiate the heterogeneity within sectors, particularly the industrial and service sectors, where activities with highly divergent carbon intensities coexist. Future research could benefit from a more granular, industry-specific exploration to design more targeted and effective decarbonization strategies.

In sum, while this study advances the understanding of CO_2_ emission determinants across income groups, addressing these limitations would enhance the precision, policy relevance, and forward-looking nature of subsequent analyses.

## Resource availability

### Lead contact

Further information and requests for resources should be directed to and will be fulfilled by the lead contact, Rihab Fannouch (rihab.fannouch@um5r.ac.ma).

### Materials availability

This study did not generate unique materials or reagents.

### Data and code availability

• All data used in this study are publicly available. The analysis is based on the World Bank World Development Indicators (WDI) for the period 1990–2023, which are openly accessible without restriction from the World Bank Open Data platform (https://data.worldbank.org/).
• All original code used to perform the machine-learning and deep-learning analyses has been deposited at Zenodo and is publicly available as of the date of publication. The DOI is listed in the key resources table.
• Any additional information required to reanalyze the data reported in this paper is available from the lead contact upon request.

## Acknowledgments

This research did not receive any specific grant from funding agencies in the public, commercial, or not-for-profit sectors.

## Author contributions

Conceptualization, R.F. and S.T.; methodology, R.F.; software, R.F.; validation, R.F. and S.T.; formal analysis, R.F.; investigation, R.F.; resources, R.F.; data curation, R.F.; writing—original draft preparation, R.F.; writing—review and editing, R.F. and S.T.; visualization, R.F.; supervision, S.T.; project administration, S.T. All authors have read and agreed to the published version of the manuscript.

## Declaration of interests

The authors declare no competing interests.

## STAR★Methods

### Key resources table

**Table:** 

REAGENT or RESOURCE	SOURCE	IDENTIFIER
**Deposited data**		

World Development Indicators (WDI), 1990–2023	World Bank	https://databank.worldbank.org/source/world-development-indicators
Original analysis code	This paper	Zenodo: https://doi.org/10.5281/zenodo.20786222

**Software and algorithms**		

Python (v3.13.5)	Python Software Foundation	https://www.python.org
scikit-learn (v1.9.0)	scikit-learn developers	https://scikit-learn.org
TensorFlow (v2.21)	TensorFlow Developers	https://www.tensorflow.org

### Experimental model and study participant details

Omitted as our study does not involve biological models or human participants. As the analysis relies exclusively on aggregate country-level indicators, an assessment of the influence of sex or gender on the results is not applicable.

### Method details

#### Data and variable selection

This research is based on annual country-level data from the World Bank’s World Development Indicators (WDI), covering the period 1990–2023. Countries are grouped into four income categories—low-, lower-middle-, upper-middle-, and high-income—according to the World Bank classification. Using the same time span for all groups provides a consistent basis for comparing emission patterns across development stages.

The selected variables capture several key dimensions of development: macroeconomic performance (GDP per capita growth and foreign direct investment), demographic dynamics (population growth), energy use (fuel imports and renewable energy consumption), trade openness (exports and imports of goods and services), sectoral structure (value added in agriculture, industry, and services), and environmental pressure (CO_2_ emissions and forest area). This multidimensional design allows for a more comprehensive assessment of how different factors are associated with CO_2_ emissions across heterogeneous national contexts. CO_2_ denotes total carbon dioxide emissions excluding land use, land-use change, and forestry (LULUCF), measured in Mt CO_2_; GDP is the annual growth rate of GDP per capita; FDI is net foreign direct investment inflows as a percentage of GDP; EXP and IMP are exports and imports of goods and services as a share of GDP; FUEL is fuel imports as a percentage of merchandise imports; POP is the population growth rate; FOR is forest area as a share of total land area; and AGV, INV, and SRV are the value-added contributions of the agriculture, industry (including construction), and services sectors, respectively, expressed as percentages of GDP. All indicators are used in the units reported in the original database.

#### Data screening and inclusion

Data screening and inclusion were performed separately within each income group, consistent with the fact that all Random Forest, correlation, and deep-learning models are estimated per income group rather than on a pooled dataset. A complete-case (list-wise) inclusion rule was applied on the eleven model variables: any country–year observation with a missing value on any of these variables was removed, and a country was retained in its income group if it contributed at least one fully observed year. Among the retained countries, the average share of non-missing years per variable exceeds 92% in every income group, indicating that retained countries are well covered rather than marginally observed. This criterion retained 18, 43, 43, and 60 countries in the low-, lower-middle-, upper-middle-, and high-income groups, respectively (296, 912, 1,042, and 1,492 country–year observations). Excluded countries were almost exclusively fragile or conflict-affected states with sparse WDI coverage (e.g., Afghanistan, Central African Republic, Eritrea, Liberia, Somalia, South Sudan) and small island states or micro-territories with incomplete reporting (e.g., Andorra, Monaco, Gibraltar, Macao SAR, and the Channel Islands).

Prior to model estimation, the dataset was additionally screened for internal consistency and extreme outliers. Complete-case analysis was adopted rather than imputation in order to avoid introducing synthetic values into a comparative, income-group-specific design, so that every model was estimated on an identical and fully observed set of inputs. All continuous variables were normalized to reduce scale effects and to stabilize the training of the deep-learning architectures. Normalization parameters were estimated on the training partition only and then applied to the validation and test partitions, so that information from future periods does not influence the scaling of the training data.

#### Modeling framework

This study adopts an integrated framework combining ensemble machine learning and deep learning to identify the main predictors of CO_2_ emissions across income groups. Gradient Boosting (GB) and Random Forest (RF) are used to capture potential non-linear relationships among macroeconomic, energy, demographic, and trade variables, with RF additionally supporting feature-importance analysis for interpretability. In parallel, deep-learning architectures, Multilayer Perceptron (MLP), Recurrent Neural Networks (RNN), and Long Short-Term Memory (LSTM) are implemented to model complex patterns and potential temporal dependence in the data. The deep-learning models are employed to forecast the evolution of the aggregate CO_2_-related indicator, thereby capturing the nonlinear mapping from macroeconomic conditions to emission outcomes, while the Random Forest model provides an interpretable ranking of the macroeconomic factors most strongly associated with emissions across income groups. All experiments were conducted in Python using the scikit-learn and TensorFlow libraries. Detailed algorithmic formulations and training derivations are provided in Data S1 (Methods S1). Throughout, variable-importance and correlation results are interpreted as predictive associations rather than causal effects.

#### Tree-based ensemble models

Tree-based ensemble models (Gradient Boosting and Random Forest) were used to quantify predictive associations between CO_2_ emissions and the selected explanatory variables, allowing for non-linear effects and interactions.^73^ GB served as a comparative ensemble baseline, whereas RF was additionally leveraged for interpretability through feature-importance analysis. Gradient Boosting builds an additive predictive model by sequentially combining weak learners, typically shallow regression trees; at each iteration, an additional tree is fitted to the current residuals (the negative gradient of the loss), enabling progressive improvement while capturing non-linear effects and interactions. Random Forest, in contrast, constructs a large collection of decorrelated regression trees trained independently on randomized subsets of the data; the randomness introduced through bootstrap sampling and random feature selection enhances robustness, reduces the risk of overfitting, and improves generalization in high-dimensional and noisy datasets. Both models were implemented in Python using scikit-learn, and key hyperparameters were selected through standard grid-search optimization. Detailed algorithmic formulations are provided in Data S1 (Methods S1).

#### Deep-learning architectures

Three deep-learning architectures were implemented to address different aspects of the data structure. The Multilayer Perceptron (MLP) acts as a benchmark feedforward network, consisting of an input layer, one or more fully connected hidden layers, and an output layer, and is able to capture static, non-temporal relationships between macroeconomic indicators and emission outcomes. Recurrent Neural Networks (RNNs) incorporate recurrent connections that allow information to persist across time steps, making them suitable for describing short-term temporal dependencies in sequential data. Long Short-Term Memory (LSTM) networks extend RNNs with memory blocks composed of memory cells and three regulating gates (input, forget, and output) governed by sigmoid activation functions; this gating mechanism allows the network to retain information across extended time steps and mitigates the vanishing-gradient problem that limits simple RNNs over long sequences. Together, these architectures provide a comprehensive approach to modeling the dynamic relationships associated with CO_2_ emissions across diverse economic contexts. Detailed formulations, architectural equations, and schematic representations are provided in Data S1 (Methods S1) and Figures S1–S3.

#### Hyperparameter tuning and setup

An extensive hyperparameter search was conducted for the deep-learning models. The number of time steps was varied across 5, 10, 15, and 20 to investigate the effect of sequence length on the ability to capture temporal dependencies; hidden-layer sizes of 32, 64, 128, 256, and 512 were evaluated to examine how network behavior changes as model complexity increases; batch sizes of 16, 32, and 64 were tested to assess their effect on training dynamics; and two optimizers, Adam and RMSprop, were compared. The same search space was explored for each of the three models (MLP, RNN, and LSTM) as a full grid search over all combinations, giving 4 × 5 × 3 × 2 = 120 candidate configurations per model, each of which was trained and evaluated. The configuration minimizing the validation loss was selected for each model, with early stopping on the validation set used to limit overfitting. The remaining training hyperparameters that were not part of the grid. namely the learning rate, the maximum number of training epochs, and the early-stopping patience were kept at the default values of the implementation and held constant across all models and configurations, so that performance differences are attributable to the searched hyperparameters rather than to differences in the training setup.

The dataset was divided into three subsets: 80% for training, 15% for validation, and 5% for testing. This 80/15/5 partition is performed along the time axis rather than by random shuffling: within each country, the earliest years are assigned to training, the following years to validation, and the most recent years to testing, so that the test set is strictly posterior to the training period and no future observation is used to predict a past one. The sliding-window sequences underlying the recurrent models (the time steps of 5–20 years) are likewise constructed within each country in chronological order, with the train/validation/test boundaries placed between dates, so that no individual sequence straddles the split and no temporal overlap occurs across subsets. The final model was then evaluated on a fully held-out test set to provide an unbiased estimate of generalization performance.

#### Out-of-time forecasting

The forecasting exercise was conducted using the Multilayer Perceptron (MLP) architecture, based on historical data extending up to 2023, with the same 80/15/5 temporal partition used for model development and evaluation. Because the models are trained on observations up to 2023 and then used to project the subsequent year (2024), this forecasting exercise is intrinsically out-of-time and provides a direct, leakage-free assessment of the models’ ability to generalize to future periods. Forecasts were generated separately for each income group.

### Quantification and statistical analysis

#### Model evaluation metrics

Classification performance was evaluated using four standard metrics: precision, recall, F1-score, and the area under the ROC curve (AUC). Precision quantifies the proportion of correctly predicted positive instances among all instances classified as positive and reflects the ability to avoid false positives. Recall (sensitivity) measures the proportion of actual positive cases correctly identified by the model and is especially critical when the cost of false negatives is high. The F1-score is the harmonic mean of precision and recall, providing a balanced metric that is particularly informative under class imbalance. The AUC-ROC evaluates the ability to distinguish between classes across a range of decision thresholds: a value close to 1 indicates strong discriminatory ability, whereas a value below 0.5 indicates performance worse than random classification. Interpreting these indicators jointly yields a robust, threshold-independent assessment of model behavior.

#### Cross-validation and significance

All ensemble models were trained and evaluated using 10-fold cross-validation. To respect the temporal structure of the data, the cross-validation procedure preserves chronological ordering: each validation fold corresponds to a period that is subsequent to the data used for training in that fold, so that model evaluation reflects out-of-time prediction rather than random resampling across years. Random Forest and Gradient Boosting performance was compared across the four income groups using AUC, F1-score, precision, and recall (reported in the Results); the model achieving the higher cross-validated performance (Random Forest) was selected for the subsequent feature-importance analysis. For the deep-learning models, the selection criterion was the validation loss, and the held-out test loss was used to compare the final MLP, RNN, and LSTM configurations. Feature-importance scores were computed from the final Random Forest models estimated separately for each income group, and Pearson correlation coefficients between CO_2_ emissions and each macroeconomic variable were computed within each income group. Throughout, feature-importance and correlation results are interpreted as predictive associations rather than causal effects. The number of countries and country–year observations analyzed in each income group is reported above under “Data screening and inclusion,” and statistical details for each analysis are reported with the corresponding tables and figures in the Results.
