# Supplementary material for: AI-based analysis of key predictors of CO2 emissions across income groups
Source: iScience. 2026 Jul 30;29(8):116976. doi: 10.1016/j.isci.2026.116976 (PMC13452225; doi:10.1016/j.isci.2026.116976)
Supplement: Supplementary file 1 [file mmc1.pdf]

iScience, Volume *29*

Supplemental information

AI-based analysis of key predictors of CO_2 emissions across income groups

Rihab Fannouch and Saïd Tounsi

Data S1. Methods S1: Detailed algorithmic formulations and derivations of the gradient boosting, random forest, and neural-network (MLP, RNN, LSTM) models used in the analysis, Related to STAR Methods.

Gradient Boosting (GB) Algorithm

Gradient Boosting (GB) is a sophisticated ensemble learning method that constructs a strong predictive model by iteratively combining multiple weak learners, typically shallow regression trees [73]. Each tree is trained to correct the residual errors of the aggregated predictions from the previous iterations, enabling the model to progressively improve its performance.

In the context of regression, GB employs decision trees that are specifically designed to estimate continuous target variables rather than perform classification. The construction of these trees involves recursively partitioning the dataset into increasingly smaller subsets. Initially, all data points are grouped together, and the algorithm evaluates every possible split across all predictors. The optimal split is selected based on the minimization of the residual error, often measured using the MSE criterion. The chosen feature is the one that most effectively separates the observations into homogeneous subsets in terms of the target variable.

At each iteration, a new tree is fitted to the residuals and added to the ensemble with a weight governed by a predefined learning rate. This controlled contribution helps reduce the risk of overfitting. The process is repeated until a specified number of trees is reached or further improvements in model accuracy become negligible. Through this iterative refinement, GB models achieve high predictive performance and are well-suited for capturing complex, non-linear relationships in structured data.

The Gradient Boosting algorithm, as formalized by Friedman (2001), operates on a set of input data $[(x_i, y_i)]_{i=1}^{n}$, and relies on a differentiable loss function $L(y_i, F(x))$ to iteratively minimize prediction error. This framework enables the model to refine its predictions through successive approximations, using gradient-based optimization techniques at each stage of the learning process.

Step 1: Initialization

The algorithm begins by defining an initial prediction function $F_0(x)$ that minimizes the overall loss across all observations. This is achieved by selecting a constant value that best approximates the response variable:

$$F_0(x) = arg\ min_\gamma \sum_{i=1}^{n} L(Y_i, \hat{y}) \quad (1)$$

Here, Y_i represents the true (observed) value and ŷ the predicted value. For the squared error loss, this corresponds to the mean of the target variable. This step provides a baseline from which subsequent refinements are made.

Step 2: Iterative Boosting Process (for m = 1 to M)

At each iteration mmm, the model updates its prediction function by fitting a new regression tree to the residuals of the previous iteration. The key steps are as follows:

- Compute the negative gradient (pseudo-residuals) of the loss function with respect to the current $F_{m-1}(x_i)$ prediction for all training samples:

$$\hat{y}_{im} = - \left[\frac{\partial L\ (Y_i,\ F\ (x_i))}{\partial F\ (x_i)}\right]_{F\ (x)=\ F_{m-1}(x)} \quad for\ i = 1, \ldots, n \quad (2)$$

- Fit a regression tree to the pseudo-residuals $\hat{y}_{im}$, and partition the input space into J_m disjoint regions R_{jm} , where j = 1, ..., J_m.
- For each region R_{jm} , determine the optimal value to minimize the loss function:

$$\hat{y}_{jm} = arg\ \min_{\gamma} \sum_{x_i \in R_{jm}} L\ (Y_i\ , F_{m-1}(x_i)\ +\ \gamma\ ) \quad (3)$$

- Update the model by adding the weighted predictions of the new tree:

$$F_m\ (x) = F_{m-1}\ (x) + \alpha \sum_{j=1}^{J_m} \hat{y}_{jm}\ .\ I\ (x\ \in\ R_{jm}) \quad (4)$$

Here, α denotes the learning rate, which controls the contribution of each tree to the overall model. A smaller learning rate slows down the learning process, enabling more refined adjustments and reducing the risk of overfitting. This regularization mechanism, combined with iterative learning, enhances both the accuracy and generalizability of the model [74].

Step 3: Final Model Output

$$\hat{F}\ (x) = F_M\ (x) \quad (5)$$

Upon completion of all M iterations, the function $\boldsymbol{F_M\ (x)}$ represents the final predictive model. This function aggregates the contributions of all preceding base learners and provides the final approximation $\boldsymbol{\hat{F}\ (x)}$ of the underlying relationship between the input features and

the target variable. The model is thus capable of capturing complex, non-linear patterns in the data with enhanced accuracy and generalizability.

The Random Forest (RF) Algorithm

The Random Forest (RF) algorithm is a robust and widely adopted ensemble learning technique in the field of machine learning [75]. Unlike boosting methods that sequentially correct errors of prior models, RF constructs a large collection of decorrelated regression trees trained independently on randomized subsets of the data. This independence and randomness introduced through bootstrap sampling and random feature selection enhance the model's robustness and reduce the risk of overfitting.

This dual-randomization strategy significantly improves generalization, particularly in high-dimensional and noisy datasets [76]. By aggregating the outputs of multiple independent learners, Random Forest achieves high predictive accuracy while maintaining scalability and resilience to variance.The fundamental steps of the RF model are outlined below:

Step 1: Tree Construction via Bootstrapping and Feature Randomization

The construction of a Random Forest involves training M individual decision trees, each on a unique bootstrapped sample of the original dataset. For each iteration m=1,…,M, the following steps are applied:

- A bootstrap sample Z of size N is drawn from the training data with replacement. This resampling introduces variance into the ensemble, which helps mitigate overfitting.

- At each node of the decision tree, a random subset of x features is selected from the total p available predictors. Among these, the optimal feature and corresponding split point are chosen to minimize the loss function typically the Mean Squared Error (MSE) in regression tasks:

$$F_0(x) = \frac{1}{n}\sum_{i=1}^{n}(Y_i - \hat{y})^2 \tag{6}$$

- Where Y_i is the actual value and $\hat{y}$ the predicted value at iteration i.

- The splitting process continues recursively, creating new branches by dividing the node into two subsets. Trees grow until a minimum node size n_{min} is reached, limiting their depth and complexity.

This dual-randomization both at the data and feature levels injects diversity across trees, which strengthens the ensemble's robustness. It reduces the likelihood that any single feature dominates the learning process and improves generalization by lowering the correlation between individual trees. Moreover, to avoid overfitting caused by overly complex trees, some implementations may prune or limit trees by discarding certain nodes or branches deemed non-contributory [77].

Step 2: Aggregation of Ensemble Predictions

Once all trees are trained, the final prediction for a given input x is computed by averaging the outputs of the individual trees:

$$\hat{F}_{rf}^{M}(x) = \frac{1}{M} \sum_{m=1}^{M} T_m(x) \tag{7}$$

This ensemble averaging stabilizes the overall prediction by reducing variance, and contributes to the high accuracy and resilience of the RF model. The resulting predictions benefit from the combined strengths of individual trees while mitigating their weaknesses, making Random Forest particularly effective for structured regression problems in the presence of noise and multicollinearity.

Neural network architectures (MLP, RNN, LSTM): equations and derivations.

Multilayer Perceptron (MLP) Neural Network

In this study, the Multilayer Perceptron (MLP) serves as the core modeling approach. As a feedforward network, the MLP consists of an input layer, one or more hidden layers, and an output layer. Each layer is fully connected to the next, forming a deep hierarchical architecture capable of complex representation learning. The overall network structure adopted in this work is illustrated in Figure S1.

Let G denote the number of hidden layers and $n_0, n_1, \ldots, n_{G+1}$ represent the number of neurons in each respective layer. The input i_n and output o_n of the n-th neuron in the first hidden layer are computed as follows:

$$i_n = \sum_{j=1}^{n_0} w_{jn}\ f_0(x_j) + b_n \quad (8)$$

$$o_n = f_1\ (i_n) = (1 + e^{-i_n})^{-1} \quad (9)$$

where x_i denotes the j-th input variable, w_{jn} is the weight connecting input neuron j to hidden neuron n, b_n is the bias term, and $f_k(\cdot)$ represents the activation function at layer k.

Similarly, the final output layer computes:

$$i_1 = \sum_{j=1}^{n_G} w_{j1} O_j + b_1 \quad (SEQ\ Equation \backslash * \ ARABIC\ 11)$$

$$o_1 = i_1 \quad (10)$$

where o_j is the output of the j-th neuron in the last hidden layer, w_{j1} is the corresponding weight, and b_1 is the output layer bias.

Most methods for training Multilayer Perceptrons (MLPs) depend on the standard error backpropagation algorithm, which seeks to iteratively adjust network parameters by reducing the difference between the network's predictions and actual results. A mini-batch gradient descent strategy is used for optimization, balancing the computational efficiency of stochastic approaches with the stability of full-batch methods. This method helps to achieve increased convergence speed and overall robustness; especially in complex, high-dimensional learning tasks it performs even better still.

To reduce overfitting and facilitate generalization, an early stopping mechanism is used. This ends the training once the model stops improving on a separate validation set. The Mean Squared Error (MSE), which is defined as: MSE = $\frac{1}{n}\sum_{i=1}^{n}(Y_i - \widehat{Y_i})^2$, is utilized to evaluate the accuracy of the model. In order to provide stable dynamic convergence during training, the initialization of the weights is carried out at random from a Gaussian distribution. This set-up enables those non-linear relationships to be modelled effectively and keeps the degree of complexity under control.

Figure S1. MLP neural network architecture with N inputs, G hidden layers, a univariate output and weights w_{jn}, Related to STAR Methods.

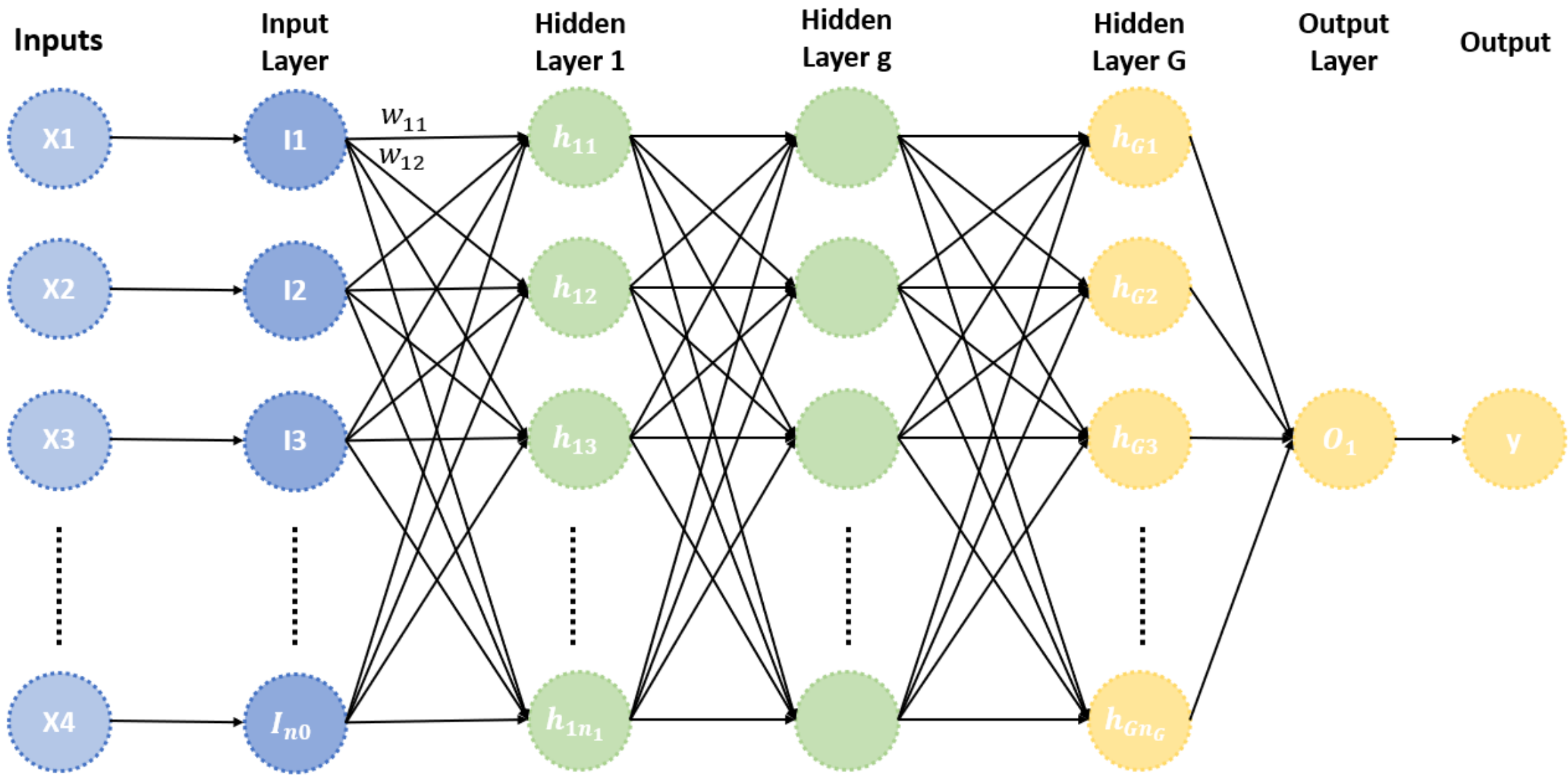

Recurrent Neural Networks (RNN)

Recurrent Neural Networks (RNNs) constitute a class of artificial neural networks specifically designed for modeling sequential or time-dependent data. Unlike traditional feedforward neural networks, RNNs incorporate recurrent connections that allow information to persist across time steps. This structural feedback loop enables RNNs to retain contextual memory, making them particularly effective for capturing temporal dependencies in complex datasets, including those involving economic and environmental variables that evolve over time.

An RNN is typically composed of three main components: an input layer, one or more hidden layers, and an output layer. Each hidden unit receives the input at the current time step along with the output from the previous hidden state, allowing the model to learn dynamic patterns across sequences. Figure S2 represents a simple RNN architecture with two hidden layers. Mathematically, given an input sequence X = $(x_1, x_2, \ldots, x_T)$, the hidden state h_t and output y_t at each time step t are computed as follows:

$$h_t = \sigma\,(W_{xh}\,x_t + W_{hh}\,h_{t-1} + b_h) \tag{11}$$

$$y_t = W_{hy}\,h_t + b_y \tag{12}$$

where σ is a nonlinear activation function (commonly tanh or ReLU), W denotes the weight matrices, and b the bias terms. The hidden state h_t encapsulates both the influence of the current input and the memory of past states, enabling the network to model sequential dependencies over time.

Typically, RNNs are trained using gradient-based optimization techniques such as back-propagation through time (BPTT), which unfolds the network over time steps and computes gradients over the whole sequence. However, the standard RNN has a well-known shortcoming: vanishing or exploding gradient problems. When learning long-range dependencies and gradients with respect to them, they can either diminish or grow exponentially, which can hinder effective weight updates and lead to unstable training.

In order to overcome these challenges, several advanced variants, such as Long Short Term Memory (LSTM) networks, have been developed . These architectures incorporate gating mechanisms that regulate information flow and help preserve gradient stability over long sequences. LSTM-RNN models are particularly beneficial in domains where latent patterns emerge over extended temporal horizons, such as in macroeconomic forecasting and the analysis of CO_2 emissions influenced by policy and trade dynamics.

In evaluating model performance, particularly in classification tasks, metrics such as the Receiver Operating Characteristic (ROC) curve and the Area Under the Curve (AUC) are employed. The ROC curve plots the true positive rate against the false positive rate across various thresholds, while the AUC quantifies the model's ability to distinguish between classes. A higher AUC value indicates greater discriminatory power. These metrics are essential for assessing the robustness and generalizability of RNN-based models applied to economic–environmental systems.

Figure S2. RNN neural network architecture with N inputs, G hidden layers, a univariate output, and recurrent connections, Related to STAR Methods.

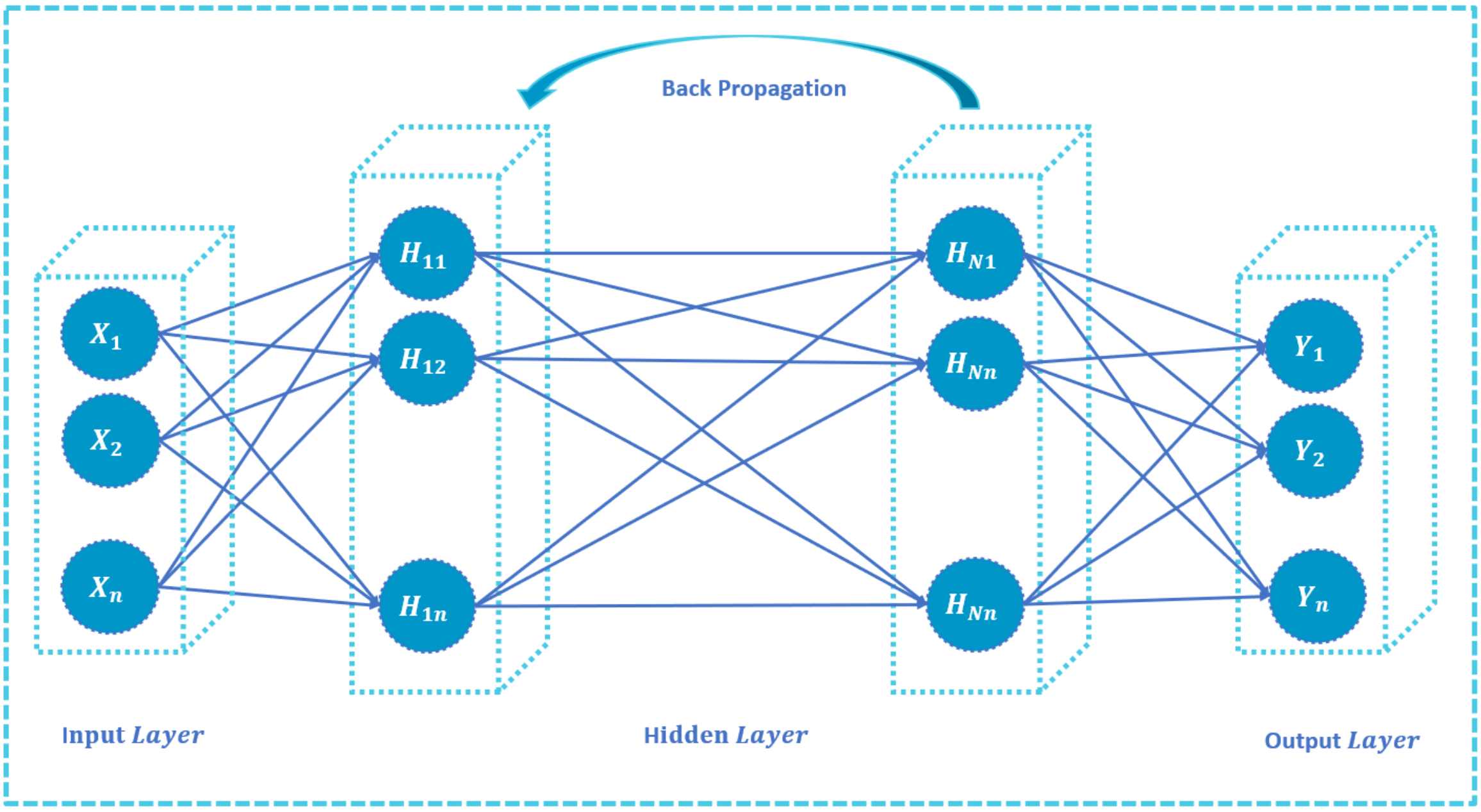

Long Short-Term Memory (LSTM)

Long Short-Term Memory (LSTM) networks are a specialized type of Recurrent Neural Network (RNN) designed to overcome the limitations of traditional RNNs, particularly the vanishing gradient problem that hinders learning over long sequences. Unlike standard RNN units, which tend to lose information over time, LSTM cells incorporate a memory mechanism that allows them to retain information across extended time steps, sometimes spanning over a thousand discrete intervals .

Each LSTM unit replaces the traditional hidden neurons with memory blocks composed of one or more memory cells and three regulating gates input, forget, and output that control the flow of information. The forget gate determines which information should be discarded from the cell state, the input gate decides which new information is stored, and the output gate regulates the exposure of the internal memory to the next layer. These gates are typically governed by sigmoid activation functions, ensuring values range between 0 and 1.

The output $y_j^f(t)$ of an LSTM memory cell at time t is calculated as:

$$y_j^f(t) = y_j^{out}(t) \, . \, h(s_j^f(t)) \tag{13}$$

Where $y_j^{out}(t)$ is the activation of the output gate, $s_j^f(t)$ is the internal state of the memory cell, and h is a nonlinear activation function such as the hyperbolic tangent applied to the cell state.

Figure S3 illustrates the internal structure of a basic LSTM cell, showing the interaction between gates and memory flow.

Figure S3. LSTM cell architecture illustrating the input gate, forget gate, output gate, and memory cell state transitions, Related to STAR Methods.

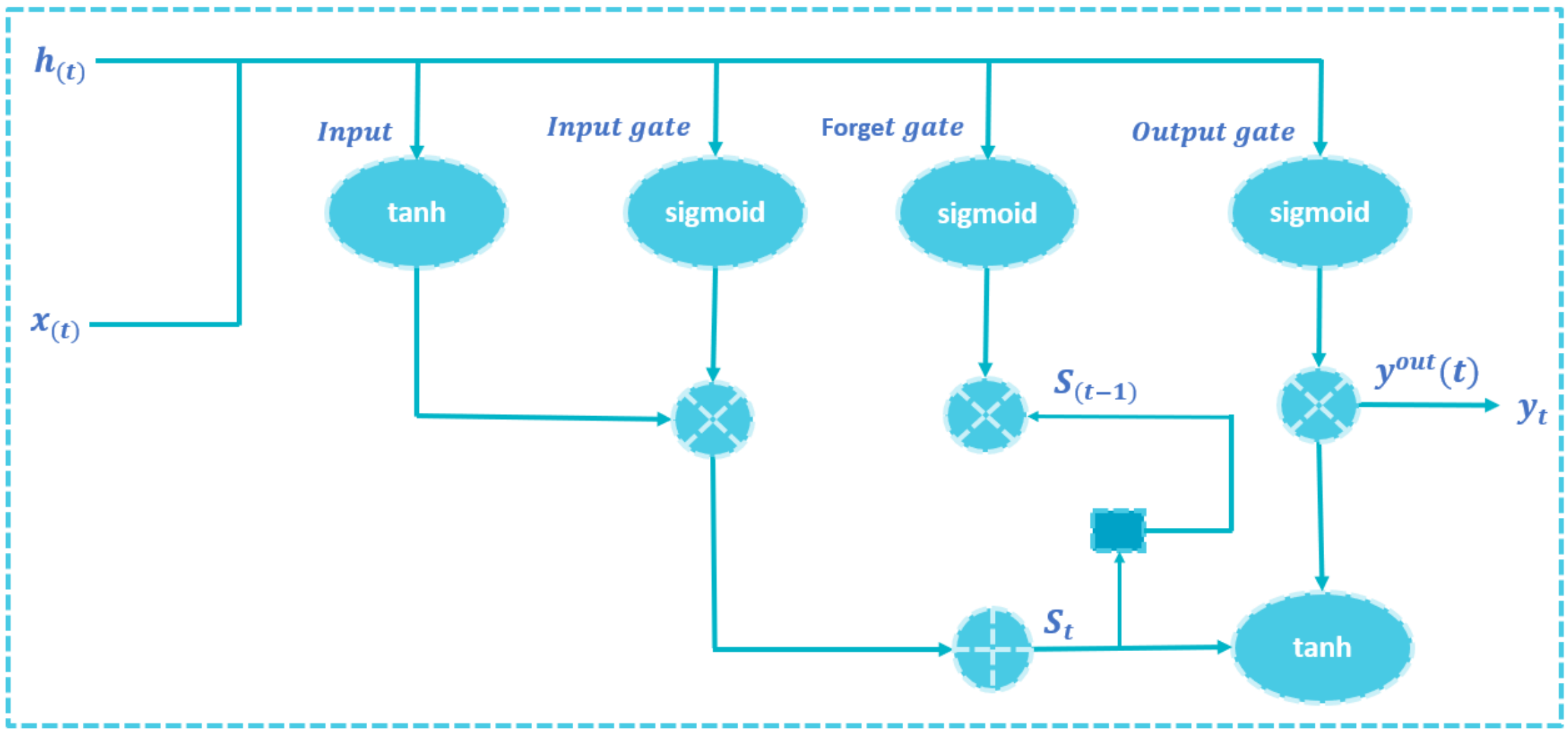

This architecture enables LSTMs to model long-range dependencies and dynamic patterns in sequential data more effectively than conventional RNNs, making them particularly well-suited for applications such as anomaly detection, time series forecasting, and natural language processing.
